# Disrupted hypothalamic transcriptomics and proteomics in a mouse model of type 2 diabetes exposed to recurrent hypoglycaemia

**DOI:** 10.1007/s00125-023-06043-x

**Published:** 2023-11-28

**Authors:** Judit Castillo-Armengol, Flavia Marzetta, Ana Rodriguez Sanchez-Archidona, Christian Fledelius, Mark Evans, Alison McNeilly, Rory J. McCrimmon, Mark Ibberson, Bernard Thorens

**Affiliations:** 1 Novo Nordisk A/S, Måløv, Denmark; 2 Center for Integrative Genomics (CIG), University of Lausanne, Lausanne, Switzerland; 3 Vital-IT Group, SIB Swiss Institute of Bioinformatics, Lausanne, Switzerland; 4 IMS Metabolic Research Laboratories, Addenbrookes Biomedical Campus, Cambridge, UK; 5 School of Medicine, University of Dundee, Dundee, UK

**Keywords:** Astrocytes, Counterregulation, Glucagon, Hypoglycaemia, Hypothalamus, Insulin, Neurodegeneration, Neurons, Oligodendrocytes, RNA-seq

## Abstract

**Aims/hypothesis:**

Repeated exposures to insulin-induced hypoglycaemia in people with diabetes progressively impairs the counterregulatory response (CRR) that restores normoglycaemia. This defect is characterised by reduced secretion of glucagon and other counterregulatory hormones. Evidence indicates that glucose-responsive neurons located in the hypothalamus orchestrate the CRR. Here, we aimed to identify the changes in hypothalamic gene and protein expression that underlie impaired CRR in a mouse model of defective CRR.

**Methods:**

High-fat-diet fed and low-dose streptozocin-treated C57BL/6N mice were exposed to one (acute hypoglycaemia [AH]) or multiple (recurrent hypoglycaemia [RH]) insulin-induced hypoglycaemic episodes and plasma glucagon levels were measured. Single-nuclei RNA-seq (snRNA-seq) data were obtained from the hypothalamus and cortex of mice exposed to AH and RH. Proteomic data were obtained from hypothalamic synaptosomal fractions.

**Results:**

The final insulin injection resulted in similar plasma glucose levels in the RH group and AH groups, but glucagon secretion was significantly lower in the RH group (AH: 94.5±9.2 ng/l [*n*=33]; RH: 59.0±4.8 ng/l [*n*=37]; *p*<0.001). Analysis of snRNA-seq data revealed similar proportions of hypothalamic cell subpopulations in the AH- and RH-exposed mice. Changes in transcriptional profiles were found in all cell types analysed. In neurons from RH-exposed mice, we observed a significant decrease in expression of *Avp*, *Pmch* and *Pcsk1n*, and the most overexpressed gene was *Kcnq1ot1*, as compared with AH-exposed mice. Gene ontology analysis of differentially expressed genes (DEGs) indicated a coordinated decrease in many oxidative phosphorylation genes and reduced expression of vacuolar H^+^- and Na^+^/K^+^-ATPases; these observations were in large part confirmed in the proteomic analysis of synaptosomal fractions. Compared with AH-exposed mice, oligodendrocytes from RH-exposed mice had major changes in gene expression that suggested reduced myelin formation. In astrocytes from RH-exposed mice, DEGs indicated reduced capacity for neurotransmitters scavenging in tripartite synapses as compared with astrocytes from AH-exposed mice. In addition, in neurons and astrocytes, multiple changes in gene expression suggested increased amyloid beta (Aβ) production and stability. The snRNA-seq analysis of the cortex showed that the adaptation to RH involved different biological processes from those seen in the hypothalamus.

**Conclusions/interpretation:**

The present study provides a model of defective counterregulation in a mouse model of type 2 diabetes. It shows that repeated hypoglycaemic episodes induce multiple defects affecting all hypothalamic cell types and their interactions, indicative of impaired neuronal network signalling and dysegulated hypoglycaemia sensing, and displaying features of neurodegenerative diseases. It also shows that repeated hypoglycaemia leads to specific molecular adaptation in the hypothalamus when compared with the cortex.

**Data availability:**

The transcriptomic dataset is available via the GEO (http://www.ncbi.nlm.nih.gov/geo/), using the accession no. GSE226277. The proteomic dataset is available via the ProteomeXchange data repository (http://www.proteomexchange.org), using the accession no. PXD040183.

**Graphical Abstract:**

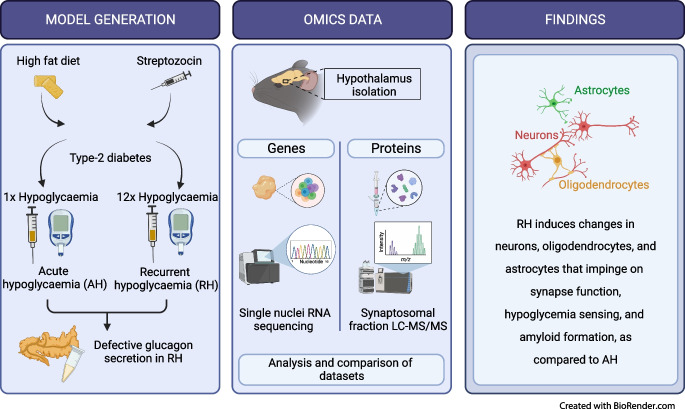

**Supplementary Information:**

The online version of this article (10.1007/s00125-023-06043-x) contains peer-reviewed but unedited supplementary material.


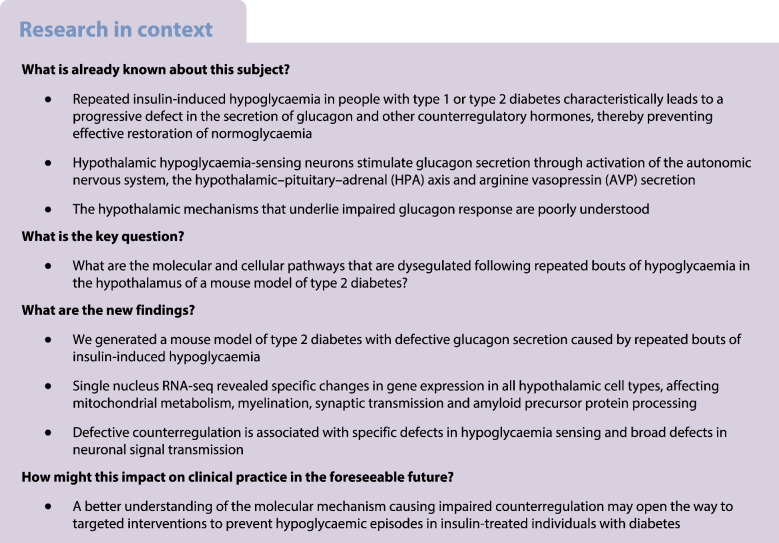


## Introduction

Repeated episodes of insulin-induced hypoglycaemia in individuals with type 1 or type 2 diabetes lead to defective secretion of glucagon and of the other counterregulatory hormones, adrenaline (epinephrine), noradrenaline (norepinephrine), cortisol and growth hormone [1]. Secretion of glucagon, which forms the first line of defence against hypoglycaemia, is controlled by multiple pancreatic-alpha-cell autonomous and non-autonomous mechanisms [2]. The role of central hypoglycaemia sensing in triggering glucagon secretion is well documented [3]. Neurons present in the brainstem, in particular in the dorsovagal complex, the parabrachial nucleus and the basolateral medulla, have been implicated in hypoglycaemia-induced glucagon secretion [4]. In the hypothalamus, glucose-sensing neurons activated by hypoglycaemia have been identified in the arcuate and in the paraventricular, the lateral and the dorsomedial nuclei. These neurons control glucagon secretion through modulation of the activity of the sympathetic or parasympathetic nerves and the sympatho-adrenal axis [3, 5]. In addition, magnocellular neurons of the paraventricular and supraoptic nuclei can be activated by hypoglycaemia, leading to secretion of arginine vasopressin (AVP) in the blood. AVP can then stimulate glucagon secretion by activating the AVP1b receptor in pancreatic islet alpha cells [6]. The mechanisms of hypoglycaemia sensing by neurons (referred to as glucose-inhibited [GI] neurons) are diverse but they share the ability to respond to a decrease in glucose metabolism and ATP production. This fall in intracellular energy level activates AMP-dependent protein kinase (AMPK), which controls membrane depolarisation by directly regulating the activity of ion channels and the production of reactive oxygen species [7, 8]. Alternatively, a reduction in the activity of the Na^+^/K^+^-ATPase induces membrane depolarisation and neuron firing [9, 10]. Thus, central hypoglycaemia sensing relies on a variety of signalling pathways, and there is little information about the dysegulations that impair the counterregulatory response (CRR) in people with diabetes treated with insulin.

Here, we sought to identify cellular and molecular pathways that are dysegulated in the hypothalamus of a mouse model of diabetes with defective hormonal counterregulation owing to exposure to single or repeated hypoglycaemic episodes.

## Methods

### Animals

Seven-week-old C57BL/6N male mice (Charles River Laboratories, Saint-Germain-Nuelles, France; http://www.criver.com/products-services/find-model/c57bl6-mouse?region=3616) were housed on a 12 h light/dark cycle. All procedures were approved by the Veterinary Office of Canton de Vaud (Switzerland; licence VD 3535) (see Electronic supplementary material [ESM] Methods). Blinding was not feasible during the generation of the model; however, results were analysed in a blinded fashion whenever possible.

### High-fat diet and streptozocin treatment of mice and insulin-induced hypoglycaemia

Mice were fed a high-fat diet (HFD; 15.6% protein, 44.4% lipids, 40.3% carbohydrates; Diet 235HF, Safe, Paris, France) for 7 weeks. Subsequently, every 2 days, for approximately 10–14 days, after a 5 h fast, mice received i.p. injections of streptozocin (STZ; S0130, Sigma Aldrich, St Louis, MO, USA), twice at 35 mg/kg and once at 70 mg/kg (see ESM Methods, ‘High-fat diet and streptozocin treatment of mice’ section). Hypoglycaemia was induced by i.p. injection of insulin (Actrapid, Novo Nordisk, Bagsværd, Denmark). The recurrent hypoglycaemia (RH) group received three insulin injections per week for 4 weeks (12 injections in total). The acute hypoglycaemia (AH) group received three saline (154 mmol/l NaCl) injections per week for 3 weeks, followed by two further saline injections in week 4 plus a final injection of insulin. Plasma glucose was measured from tail-vein blood at −60 min, 60 min and 120 min after the final i.p. injection, using a glucometer (Contour XT, Bayer, Leverkusen, Germany). Plasma glucagon was measured from submandibular vein blood using an ELISA (catalogue no. 10-1271-01; Mercodia, Uppsala, Sweden) (see ESM Methods, ‘Insulin-induced hypoglycaemia and glucagon measurements’ for details).

### Nuclei isolation, single-nuclei RNA-seq and data analysis

Brains were collected and snap frozen 120 min after the last insulin i.p. injection. Hypothalamus and cortex samples were dissected and nuclei were isolated (see ESM Methods, ‘Nuclei isolation’ section for details). For hypothalamus samples, single-nuclei libraries were prepared using the Chromium Next GEM Single Cell Multiome ATAC + Gene Expression assay (PN-1000283; 10x Genomics, Pleasanton, CA, USA), whilst, for cortex samples, we used the Chromium Single Cell 3′ RNA-seq assay (PN-1000075; 10x Genomics). All libraries were sequenced on the Illumina NovaSeq 6000 (Illumina, San Diego, CA, USA) (see ESM Methods, ‘Single-nuclei RNA sequencing’ section for details). The R package ‘Seurat’ (v4.1.1) [11] was used for data normalisation, quality control (QC) and clustering analysis (see ESM Methods, ‘Single-nuclei sequence analysis’ section). Gene ontology biological process (GO-BP) analysis and enrichment analysis of Kyoto Encyclopedia of Genes and Genomes (KEGG) terms were performed using a gene-set enrichment analysis (GSEA) approach using the R package ClusterProfiler (v4.4.4) [12] (see ESM Methods, ‘Gene-set enrichment analysis’ section).

### Synaptosome proteomic analysis

Hypothalamic synaptosomal fractions were prepared following tissue homogenisation by differential centrifugation (see ESM Methods, ‘Synaptosome preparation’ section). Fractions were digested by trypsin and loaded on a TIMS-TOF Pro (Bruker, Bremen, Germany) mass spectrometer interfaced through a nanospray ion source to an Ultimate 3000 RSLCnano HPLC system (Dionex, Sunnyvale, CA, USA) (see ESM Methods, ‘Proteomics sample preparation’ and ‘LC-MS analyses’ sections). Data generation and analysis were performed as detailed in the ESM Methods, ‘Proteomics data analysis’ and ‘Bioinformatic analysis from proteomics data’ sections. All raw MS data, together with raw output tables, are available via the ProteomeXchange data repository (http://www.proteomexchange.org) with the accession PXD040183.

### Statistical analysis

Unless stated otherwise, data are expressed as mean±SEM. Statistical analysis was performed using GraphPad Prism 8.4.0 (GraphPad Software, San Diego, CA, USA), using either a mixed-effects analysis followed by Sidak's post hoc test, a repeated-measures two-way ANOVA followed by Sidak's post hoc test, or an unpaired two-tailed Student's *t* test. Bonferroni and Benjamini–Hochberg corrections were applied for multiple comparisons. All *p* values <0.05 were considered to be significant.

## Results

### Repeated hypoglycaemia in mouse models of diabetes

**Fig. 1:**
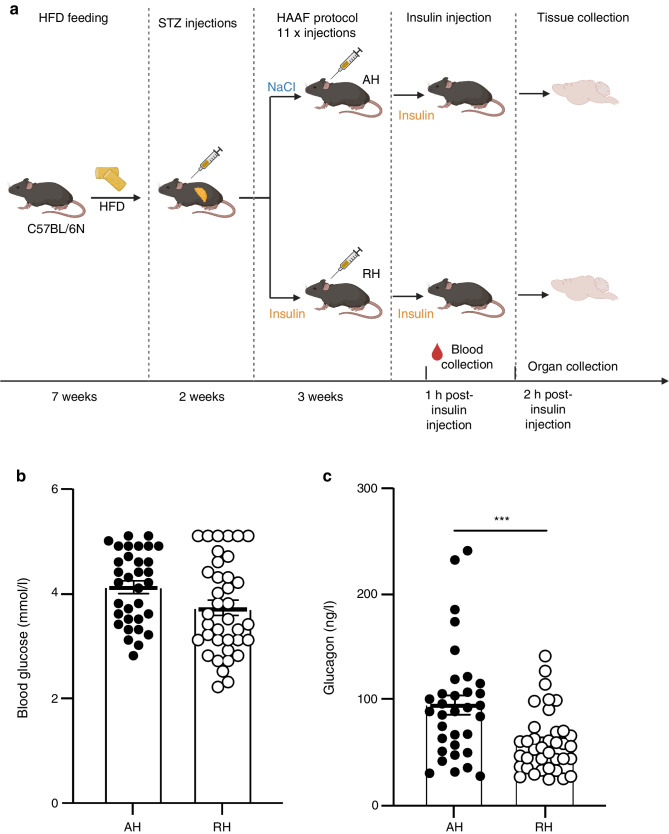
Recurrent exposure to hypoglycaemia reduces insulin-induced glucagon secretion. (**a**) Outline of the experiment to generate a model of type 2 diabetes and hypoglycaemia-associated autonomic failure (HAAF) in C57BL/6N male mice exposed to AH or RH. Created with BioRender.com. (**b**, **c**) Blood glucose (**b**) and plasma glucagon (**c**) in HFD-fed/STZ-treated mice exposed to AH or RH 1 h after a final i.p. injection of insulin (AH: *n*=33; RH: *n*=37). Data are means±SEM. ***p<0.001

C57BL/6N mice were fed an HFD for 7 weeks and then received three STZ injections, as described in the methods (Fig. 1a), to create a mouse model of chemically induced type 2 diabetes. One week after the last STZ injection, the mice became hyperglycaemic and displayed lower body weight vs pre STZ treatment, although the latter change was non-significant (ESM Fig. 1a,b). Mice were divided into two groups of identical mean body weight and plasma glucose. One group received 11 injections of saline and a final injection of insulin over a period of 4 weeks (AH group) and the other group received 12 injections of insulin over the same period of time (RH group). The blood glucose levels before and after each injection are presented in ESM Fig. 1c. Plasma glucagon levels were measured 1 h after the last insulin injection. Insulin induced a deeper hypoglycaemia in the RH group (*n*=37) than in the AH group (*n*=33) (Fig. 1b; RH: 3.7±0.1 mmol/l; AH: 4.1±0.1 mmol/l; *p*=0.05) but a lower plasma glucagon response (Fig. 1c; RH: 59.0±4.8 ng/l; AH: 94.5±9.2 ng/l; ***p<0.001).

### Single-nuclei transcriptional profiling of the hypothalamus from AH- and RH-exposed diabetic mice

**Fig. 2:**
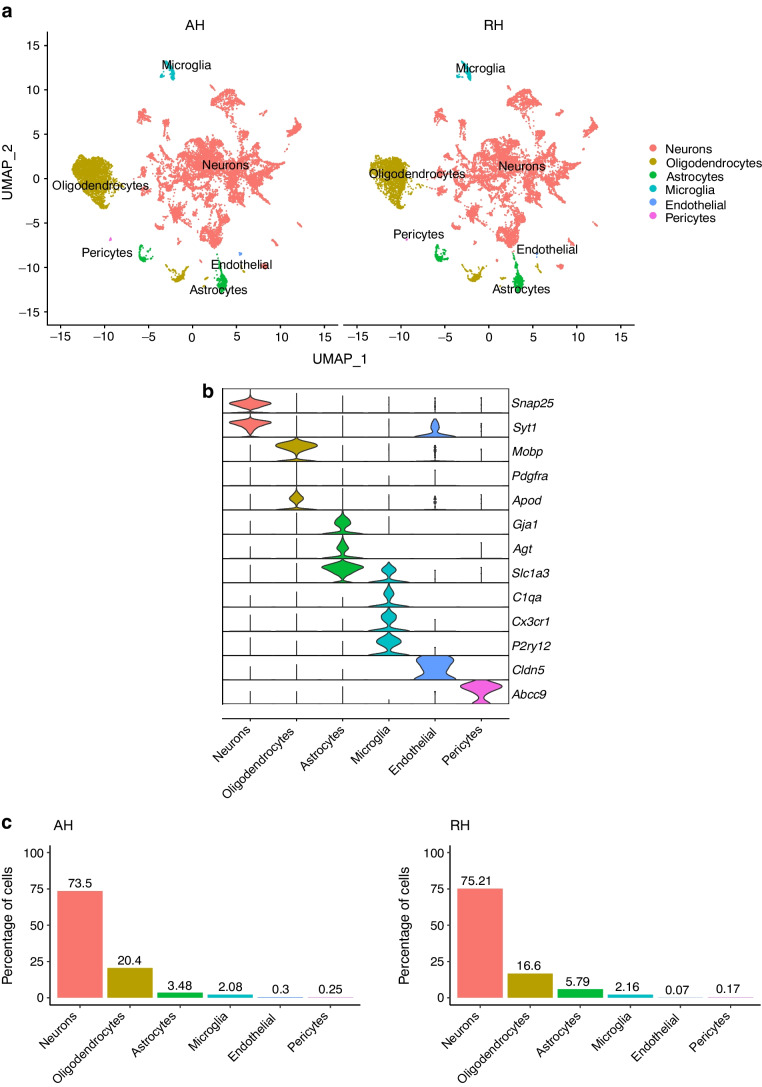
Clustering and annotation of hypothalamic snRNA-seq in HFD-/STZ-treated mice exposed to RH and AH. (**a**) Two-dimensional Uniform Manifold Approximation and Projection (UMAP-1 and UMAP-2) representation of 14,979 and 14,934 nuclei isolated from the hypothalamus of AH- and RH-exposed mice, respectively. The cells are coloured based on the annotated cell type. (**b**) Violin plots of combined data from the RH and AH groups showing the expression of cellular-specific gene markers in each of the identified cell types. (**c**) Bar plot depicting the percentages of the different hypothalamic cell types per condition. Samples from *n*=3 mice were pooled for each group and the experiment was repeated once to obtain a biological replicate (*n*=2 integrated datasets per condition)

The hypothalami from three mice in the AH group and three mice in the RH group were collected 2 h after the last insulin injection and were pooled for each group for nuclei preparations and single-nuclei RNA-seq (snRNA-seq). The experiment was repeated once to obtain a biological replicate. We computed per-cell QC metrics to identify and remove low-quality nuclei, resulting in a dataset of 14,979 nuclei from the AH group and 14,934 nuclei from the RH group (ESM Fig. 2a–e). The major brain cell types could be identified based on the expression of their characteristic gene markers (Fig. 2a,b and ESM Fig. 3a). The cell type composition was very similar in the hypothalamus of AH and RH mice (Fig. 2c), with neurons being the most abundant, followed by oligodendrocytes and astrocytes. Microglia, endothelial cells and pericytes were much less represented and will not be discussed further.

### Differential hypothalamic gene expression analysis

#### Neurons

**Fig. 3:**
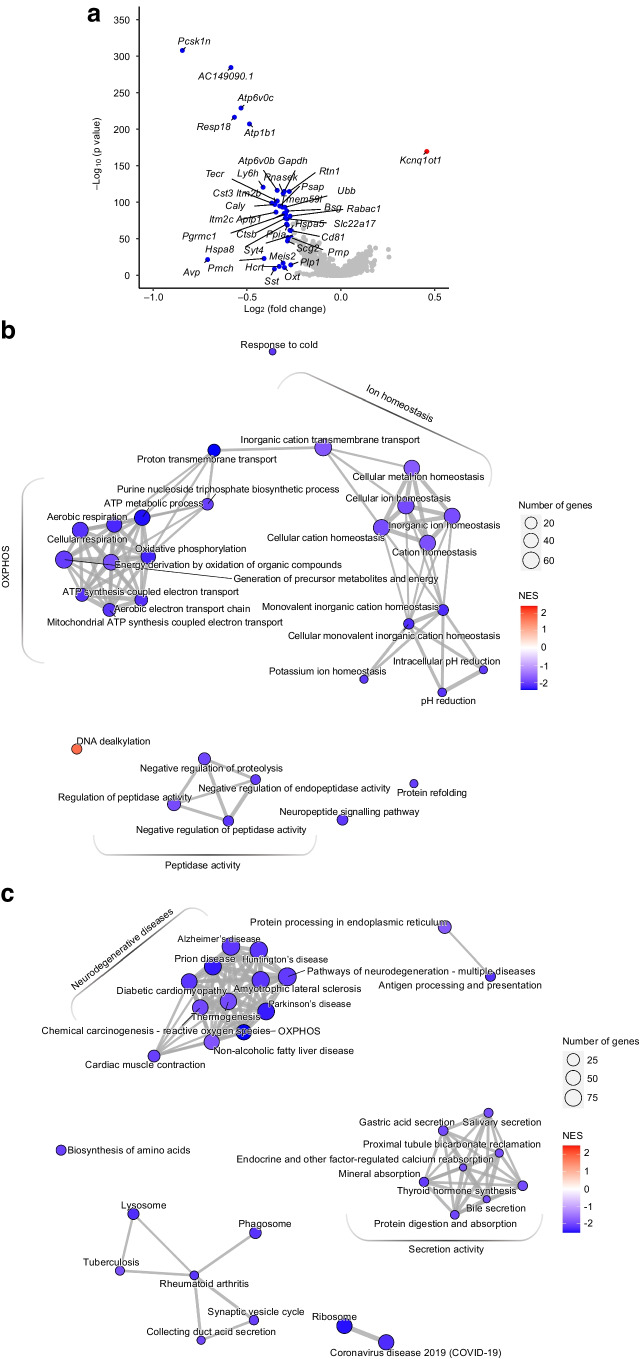
Transcriptional analysis of neurons from hypothalami of HFD-/STZ-treated mice exposed to AH or RH. Samples from *n*=3 mice were pooled for each group and the experiment was repeated once to obtain a biological replicate (*n*=2 integrated datasets per condition). (**a**) Volcano plot depicting differential expression of 14,909 genes from hypothalamic neurons of mice subjected to RH as compared with AH. The red dot outlines the upregulated DEG and blue dots outline downregulated DEGs (fold change >1.2 or <−1.2 and Bonferroni adjusted *p* value [*p*_*adj*_]<0.05). (**b**, **c**) Network visualisation of the top enriched GO-BP (**b**) or KEGG (**c**) terms in DEGs in the RH vs AH group. Node colour indicates the normalised enrichment score (NES); node size indicates the number of core-enriched genes; edges (grey lines) represent the pairwise similarity between terms

To delineate transcriptional changes occurring in the hypothalamus of mice in the RH vs AH group, we compared the transcriptional profiles for all major cell types. Analysis of neurons revealed that the majority of differentially expressed genes (DEGs) were downregulated after RH vs AH (Fig. 3a, Table 1 and ESM Table 1). Among the downregulated genes were *Pcsk1n* (an inhibitor of the proprotein convertase subtilisin/kexin type 1 [PCKS1]), *Avp* (encoding AVP, an inducer of glucagon secretion [6]) and *Pmch* (encoding pro-melanin concentrating hormone, an activator of the hypothalamic–pituitary–adrenal [HPA] axis) [13]. Other significantly downregulated genes were *Resp18* [14], *Rtn1* [15], *Psap* [16], *Ubb* [17], *Aplp1* [18], *Cst3* [19] and *Itm2b* [20], which are all associated with neurodegenerative diseases. Of note, *Kcnq1ot1* [21], a long non-coding RNA (lncRNA) involved in diabetes susceptibility, was the only overexpressed gene.

**Table 1:** DEGs with **│**fold change (FC)**│ **>1.2 identified in cells of the hypothalamus of RH- vs AH-exposed mice (Bonferroni adjusted *p* value [*p*_adj_]<0.05)

Neurons		Oligodendrocytes		Astrocytes	
Upregulated (*n*= 1)	Downregulated (*n*=38 )	Upregulated (*n*= 61)	Downregulated (*n*= 25)	Upregulated (*n*= 16)	Downregulated (*n*= 12)
*Kcnq1ot1*^a,b^	*AC149090.1*^a,b^	*Adarb2*	*AC149090.1*^b,c^	*Arap2*	*AC149090.1*^a,c^
	*Aplp1*^a^	*Adgrl3*	*Aplp1*^c^	*Atad2b*	*Apoe*
	*Atp1b1*	*Arhgap24*	*Apod*	*Gulp1*	*Atp6v0c*^c^
	*Atp6v0b*	*Brinp3*	*Apoe*	*Hdac4*	*Cd63*
	*Atp6v0c*^b^	*Chst11*	*Cd81*^b,c^	*Hdac8*	*Cd81*^a,c^
	*Avp*	*Cntn4*	*Cldn11*	*Jmjd1c*	*Clu*
	*Bsg*	*Cobl*	*Cmtm5*	*Kank1*	*Cst3*^c^
	*Caly*	*Cpq*	*Degs1*	*Kcnq1ot1*^a,c^	*Grcc10*
	*Cd81*^a,b^*)*	*Csmd2*	*Evi2a*	*Magi1*	*Itm2b*^a,c^
	*Cst3*^b^*)*	*Ctnnd2*	*Fth1*	*Meis1*	*Pcsk1n*^a,c^
	*Ctsb*	*Dcc*	*Gapdh*^c^	*Pitpnc1*	*Scd2*
	*Gapdh*^a^	*Dgkb*	*Hsp90aa1*	*Prex2*	*Scg2*^c^
	*Hcrt*	*Dlgap*	*Itm2b*^b,c^	*Rgs20*	
	*Hspa5*	*Dpyd*	*Lamp1*	*Spire1*	
	*Hspa8*	*Dscam*	*Mag*	*Tom1l1*	
	*Itm2b*^a,b^	*Fbxl7*	*Mal*	*Ttc28*	
	*Itm2c*	*Fchsd2*	*Pcsk1n*^b,c^		
	*Ly6h*	*Fut9*	*Plp1*^c^		
	*Meis2*	*Gfra1*	*Ptgds*		
	*Oxt*	*Gm4876*	*Scd2*		
	*Pcsk1n*^a,b^	*Gpc5*	*Serinc1*		
	*Pgrmc1*	*Itga9*	*Sgk1*		
	*Plp1*^a^	*Itpr2*	*St6 galnac3*		
	*Pmch*	*Kcnd2*	*Syt11*		
	*Ppia*	*Kcnq1ot1*^b,c^	*Trf*		
	*Prnp*	*Kif12*			
	*Psap*	*Lhfpl3*			
	*Rabac1*	*Lsamp*			
	*Resp18*	*Luzp2*			
	*Rnasek*	*Maml2*			
	*Rtn1*	*Map2*			
	*Slc22a17*	*Mmp16*			
	*Scg2*^b^	*Nav2*			
	*Sst*	*Nav3*			
	*Syt4*	*Ndufs4*			
	*Tecr*	*Nmnat2*			
	*Tmem59l*	*Npas3*			
	*Ubb*	*Nrxn1*			
		*Ntm*			
		*Opcml*			
		*Pcdh15*			
		*Pde7b*			
		*Ppp2r2b*			
		*Ptprt*			
		*Ptprz1*			
		*Ralyl*			
		*Robo1*			
		*Rora*			
		*Sema3d*			
		*Sntg1*			
		*Sox2ot*			
		*Sox5*			
		*Sox6*			
		*Spon1*			
		*Tafa1*			
		*Tmem132d*			
		*Tnr*			
		*Vcan*			
		*Xylt1*			
		*Zeb1*			
		*Zup1*			

^a^Also found in oligodendrocytes

^b^Also found in astrocytes

^c^Also found in neurons

We next used GSEA to interrogate the GO-BP and KEGG databases to identify the biological processes regulated by the DEGs. Several GO-BP terms downregulated in the RH group were related to ‘oxidative phosphorylation’ (OXPHOS) and ‘ion homeostasis’ (Fig. 3b). Many OXPHOS genes were core-enriched in the ‘oxidative phosphorylation’ term, and genes encoding vacuolar and plasma membrane ATPases were present in the ‘ion homeostasis’ term (ESM Table 2). KEGG analysis revealed that the most downregulated terms were related to ‘neurodegenerative diseases’, which comprise OXPHOS genes belonging to Complexes I, III and IV and ATPsynthase, and ‘secretion activity’ comprising mostly the α and β subunits of the Na^+^/K^+^-ATPase, and several subunits of the vacuolar H^+^-ATPase (V-ATPase) (Fig. 3c and ESM Table 3).

#### GABAergic vs glutamatergic neurons

To gain further insight into neuron-specific transcriptional responses to RH, we analysed the two primary neuronal classes, GABAergic and glutamatergic neurons, identified by the expression of *Gad1* and *Gad2* genes, encoding γ-aminobutyric acid (GABA)-producing enzymes, or of the vesicular glutamate transporter-encoding gene *Slc17a6*, respectively (ESM Fig. 3b). Most of the DEGs (ESM Fig. 4a,b, Table 2 and ESM Table 1) were common to both neuronal populations. However, *Avp* and *Pmch* were significantly decreased in GABAergic neurons, as were two regulators of synaptic activity, *Rabac1* [22] and *Syt11* [23]. In glutamatergic neurons, there was decreased expression of *Aldoa* (a regulator of glycolysis [24]), *Atp1a3* (encoding the α3 subunit of the Na^+^/K^+^-ATPase) and *Ptprn* (encoding protein tyrosine phosphatase N, which controls secretion of neurotransmitters [25]). There was also an increased expression of *Dgkb* (encoding diacylglycerol kinase, a regulator of dendritic outgrowth and spine maturation [26]). Thus, RH induced dysegulated expression of primarily the same genes in glutamatergic and GABAergic neurons, but with cell-specific changes in genes regulating synaptic vesicle secretion.

**Table 2:** DEGs with │fold change (FC)│ >1.2 that are commonly or exclusively identified in the different neuron types in the hypothalamus of RH- vs AH-exposed mice (Bonferroni adjusted *p* value [*p*_adj_]<0.05)

Common		Exclusive GABAergic		Exclusive glutamatergic	
Up (1)	Down (25)	Up (0)	Down (11)	Up (2)	Down (7)
*Kcnq1ot1*	*AC149090.1*		*Avp*	*Dgkb*	*Aldoa*
	*Aplp1*		*Cd81*	*Sfta3-ps*	*Atp1a3*
	*Atp1b1*		*Hspa5*		*Hspa90ab1*
	*Atp6v0b*		*Meis2*		*Plp1*
	*Atp6v0c*		*Ndn*		*Ppia*
	*Bsg*		*Pmch*		*Ptprn*
	*Caly*		*Prnp*		*Rtn1*
	*Cst3*		*Rabac1*		
	*Ctsb*		*Serinc1*		
	*Gapdh*		*Syt11*		
	*Hspa8*		*Tuba1a*		
	*Itm2b*				
	*Itm2c*				
	*Ly6h*				
	*Pcsk1n*				
	*Pgrmc1*				
	*Psap*				
	*Resp18*				
	*Rnasek*				
	*Sgc2*				
	*Slc22a17*				
	*Syt4*				
	*Tecr*				
	*Tmem59l*				
	*Ubb*				

#### Oligodendrocytes

**Fig. 4:**
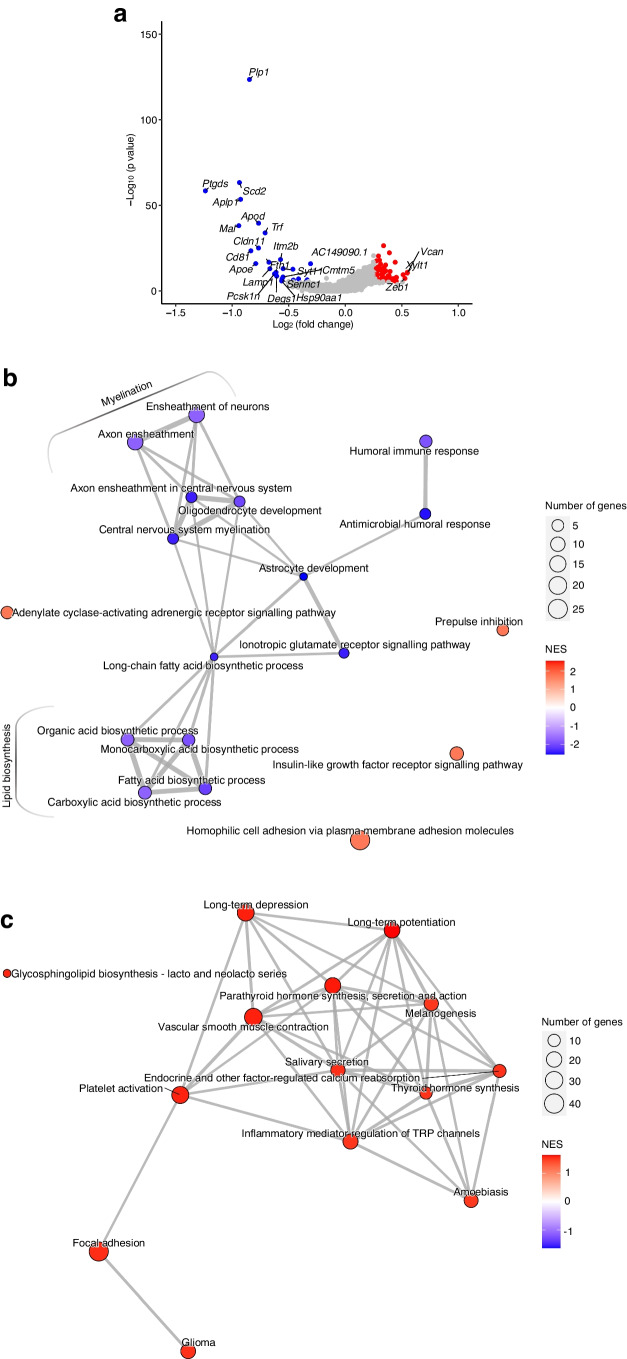
Transcriptional analysis of oligodendrocytes from hypothalami of HFD-/STZ-treated mice exposed to AH or RH. Samples from *n*=3 mice were pooled for each group and the experiment was repeated once to obtain a biological replicate (*n*=2 integrated datasets per condition). (**a**) Volcano plot depicting differential expression of 10,814 genes from hypothalamic oligodendrocytes of mice subjected to RH as compared with AH. Red dots outline upregulated DEGs and blue dots outline downregulated DEGs (fold change >1.2 or <−1.2 and Bonferroni adjusted *p* value [*p*_*adj*_]<0.05); only genes with log_2_ fold change >0.5 or l<−0.5 have been labelled. (**b**, **c**) Network visualisation of the enriched GO-BP (**b**) or KEGG (**c**) terms in DEGs in RH- vs AH-exposed mice. Node colour indicates the normalised enrichment score (NES); node size indicates the number of core-enriched genes overlapping gene count; edges (grey lines) represent the pairwise similarity between terms. TRP, transient receptor potential

The volcano plot in Fig. 4a shows that there was reduced expression of *Plp1*, *Mal*, *Scd2*, *Apoe*, *Ptgds*, *Cldn11* and *Itm2b*, which are all involved in various aspects of myelin formation [27], whilst the most overexpressed genes were *Xylt1*, *Vcan* and *Zeb1* (ESM Table 1). GSEA analysis of DEGs revealed downregulation of GO-BP terms related to the ‘lipid biosynthesis’ and ‘myelination’ pathways (Fig. 4b, ESM Table 2). KEGG analysis showed significant increases in pathways related to various intracellular signalling pathways, including phosphoinositide 3 (PI3) kinases, protein kinase C and the cAMP pathway (Fig. 4c, ESM Table 3).

#### Astrocytes

GSEA analysis of DEGs (ESM Fig. 5a, Table 1 and ESM Table 1) showed repression of GO-BP terms related to ‘lipid biosynthesis’, ‘metabolism of amyloid beta protein’ and ‘ion transport’ (ESM Fig. 5b, ESM Table 2). Worth noting is the significant down-expression of *Cst3*, *Apoe Itm2b*, *Clu* and *Atp6v0c* (ESM Fig. 5a, Table 1), all involved in protecting against neurodegenerative diseases [19, 20, 28–30]. Similarly, a KEGG functional analysis showed decreased enrichment in pathways related to ‘neurodegenerative diseases’ and increased expression of ‘signalling pathways’ (ESM Fig. 5c, ESM Table 3).

### Comparative analysis of transcriptomics and proteomic analysis

**Fig. 5:**
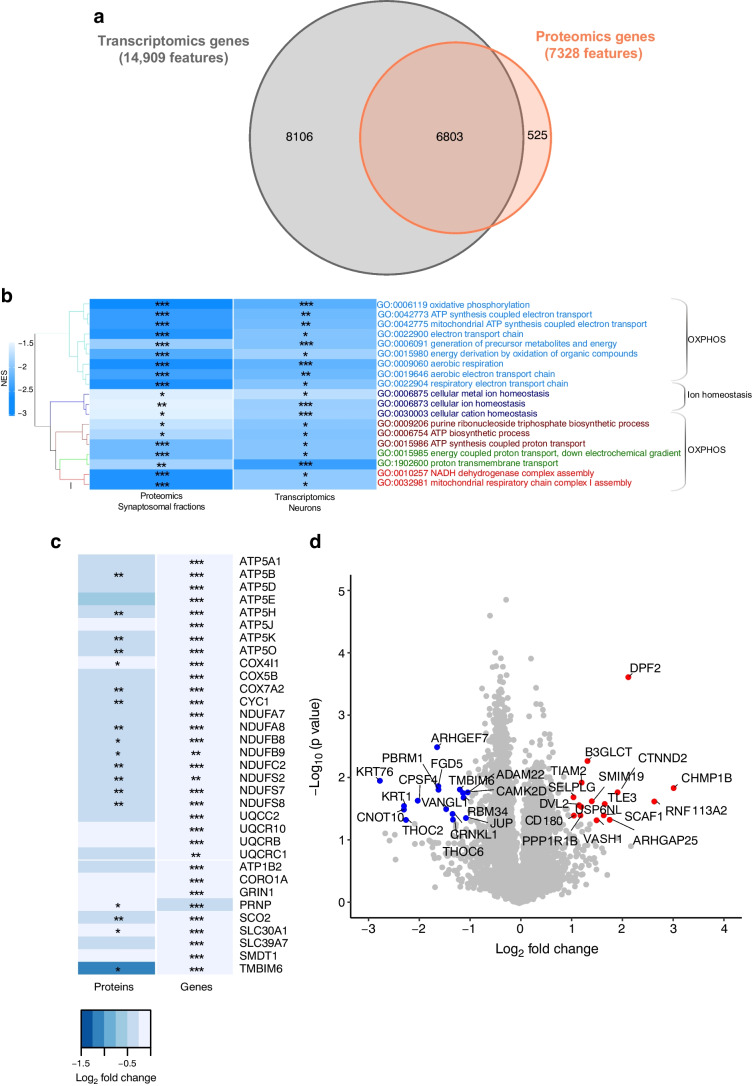
Comparison of neuronal transcriptomic and synaptosomal proteomic data from HFD-/STZ-treated mice exposed to AH or RH. Proteomics data originated from the analysis of the hypothalami of *n*=4 mice/group. (**a**) Venn diagram illustrating the number of features analysed in the proteomics analysis of synaptosomal fractions and in the transcriptomics analysis of neuronal nuclei from the hypothalamus of mice exposed to RH vs AH. (**b**) Hierarchical clustering of GO-BP terms enriched in both synaptosomal fractions and neuron transcriptomes of hypothalamus from RH- vs AH-exposed mice (|normalised enrichment score [NES]| >1.5 and Benjamini–Hochberg adjusted *p* value [*p*_adj_]<0.05). Colour of cells indicates the NES. Corresponding *p*_adj_ are also indicated (*p<0.05, **p<0.01, ***p<0.001). (**c**) Heatmap depicting log_2_ fold change in expression and *p* values (*p<0.05, **p<0.01, ***p<0.001) of selected features linked to OXPHOS or ion homeostasis. (**d**) Volcano plot depicting differential expression of 7328 proteins detected in hypothalamic synaptosomes from mice subjected to RH as compared with AH. Red and blue dots indicate proteins that are significantly differentially expressed (fold change >2 or <−2 and *p*<0.05). ATP1B2, sodium/potassium-transporting ATPase subunit beta-2; ATP5A1/ATP5B/ATP5D/ATP5E/ATP5H/ATP5K/ATP5O, mitochondrial ATP synthase subunit alpha/beta/delta/epsilon/d/e/O; ATP5J, ATP synthase-coupling factor 6, mitochondrial; CORO1A, Coronin-1A; GRIN1, glutamate receptor; COX4I1/COX5B/COX7A2, mitochondrial cytochrome *c* oxidase subunit 4 isoform 1/subunit 5B/subunit 7A2; CYC1, cytochrome c1, heme protein, mitochondrial; NDUFA7/NDUFA8, NADH dehydrogenase [ubiquinone] 1 alpha subcomplex subunit 8; NDUFB8, mitochondrial NADH dehydrogenase [ubiquinone] 1 beta subcomplex subunit 8; NDUFB9, NADH dehydrogenase [ubiquinone] 1 beta subcomplex subunit 9; NDUFC2, NADH dehydrogenase [ubiquinone] 1 subunit C2; NDUFS2/NDUFS7/NDUFS8, mitochondrial NADH dehydrogenase [ubiquinone] iron-sulfur protein 2/7/8; PRNP, major prion protein; SCO2, mitochondrial protein SCO2 homologue; SLC30A1, solute carrier family 30, member 1; SLC39A7, solute carrier family 39, member 7; SMDT1, single-pass membrane protein with aspartate rich tail 1; TMBIM6, Bax inhibitor motif containing 6; UQCC2, ubiquinol-cytochrome-c reductase complex assembly factor 2; UQCR10/UQCRB/QCRC1, cytochrome b-c1 complex subunit 9/7/1

We performed proteomic analysis of synaptosomal fractions as our transcriptomic analysis indicated that several DEGs were related to synaptic structure and activity. Proteomic profiling of synaptosomal fractions from the hypothalamus of AH- and RH-exposed mice detected 7328 proteins in total, of which 6803 had their mRNAs detected in the snRNA-seq analysis (Fig. 5a). Comparative proteomics and transcriptomics GO-BP analysis identified several common terms related to ‘oxidative phosphorylation’ and ‘ion homeostasis’ (Fig. 5b, ESM Table 4). In these GO-BP terms, many proteins displayed changes in expression that correlated with changes in their mRNA expression (Fig. 5c). This indicates that transcript profiling is in large part predictive of changes in protein expression.

Analysis of the synaptosomal proteins that showed highest differential expression between RH- and AH-exposed mice (Fig. 5d, Table 3), and for which their mRNA was not differentially expressed, revealed a relatively large group of proteins involved in synaptic remodelling [31, 32]. These included the downregulated proteins calcium/calmodulin dependent protein kinase II delta (CAMK2D), Rho guanine nucleotide exchange factor 7 (ARHGEF7), FYVE, also known as RhoGTP exchange factor and PH domain-containing protein 5 (FGD5), disintegrin and metalloproteinase domain-containing protein 22 (ADAM22) and Vang-like planar cell polarity protein 1 (VANGL1), and the upregulated proteins catenin delta-2 (CTNND2), Rho GTPase-activating protein 25 (ARHGAP25), USP6 N-terminal-like protein (USP6NL) and T cell lymphoma invasion and metastasis 2 (TIAM2) [31–36]. Other proteins found to be significantly differentially expressed are involved in mRNA splicing and export from the nucleus, including THO complex subunit 2 (THOC2), THO complex subunit 6 (THOC6) [37] and crooked neck pre-mRNA splicing factor 1 (CRNKL1) (all downregulated) and splicing factor, SR-related C-terminal domain associated factor 1 (SCAF1) (upregulated). Other upregulated proteins include the transcription factors double plant homeodomain fingers 2 (DPF2) and transducin-like enhancer family member 3, transcriptional corepressor (TLE3), and the signalling proteins segment polarity protein dishevelled segment polarity protein 2 (DVL2), protein phosphatase 1 regulatory inhibitor subunit 1B (PPP1R1B) and vasohibin 1 (VASH1). Since the mRNAs for these proteins were not found in the DEGs, this suggests that their expression is regulated at the post-translational level.

**Table 3:** Differentially expressed proteins with │fold change (FC)│ >2 identified in the synaptosomal fractions of the hypothalamus of RH- vs AH-exposed mice (*p*<0.05)

Upregulated (*n*=16)	Downregulated (*n*=16)
ARHGAP25	ADAM22
B3GLCT	ARHGEF7
CD180	CAMK2D
CHMP1B	CNOT10
CTNND2	CPSF4
DPF2	CRNKL1
DVL2	FGD5
PPP1R1B	JUP
RNF113A2	KRT1
SCAF1	KRT76
SELPLG	PBRM1
SMIM19	RBM34
TIAM2	THOC2
TLE3	THOC6
USP6NL	TMBIM6
VASH1	VANGL1

B3GLCT, beta-1,3-glucosyltransferase; CHMP1B, charged multivesicular body protein 1B; CNOT10, Ccr4–Not transcription complex subunit 10; CPSF4, cleavage and polyadenylation specific factor 4; CRNKL1, crooked neck pre-mRNA splicing factor 1; DPF2, double plant homeodomain fingers 2; DVL2, dishevelled segment polarity protein 2; JUP, junction plakoglobin; KRT1/KRT76, keratin 1 /76; PBRM1, polybromo 1; PPP1R1B, protein phosphatase 1 regulatory inhibitor subunit 1B; RBM34, RNA binding motif protein 34; RNF113A2, ring finger protein 113A2; SCAF1, SR-related C-terminal domain associated factor 1; SELPLG, selectin P ligand; SMIM19, small integral membrane protein 19; THOC2/THOC6, THO complex subunit 2/6; TLE3, transducin-like enhancer family member 3, transcriptional corepressor; TMBIM6, transmembrane Bax inhibitor motif containing 6; VANGL1, Vang-like planar cell polarity protein 1; VASH1, vasohibin 1

### Single-nuclei transcriptional profiling of the cortex from AH and RH mice

To determine whether the impact of RH on the hypothalamus was specific for this brain structure, we repeated the snRNA-seq analysis in duplicate using nuclei from the cortex of RH- and AH-exposed mice. A total of 4650 and 9088 nuclei were obtained from the AH and RH groups, respectively (ESM Fig. 6a), and QC analysis was performed as for the hypothalamus (ESM Fig. 6b-e). Cluster annotation led to the identification of the same six cell types and similar relative distribution of these cells to that observed in the hypothalamus (ESM Fig. 7 and ESM Fig. 8a–c).

**Fig. 6:**
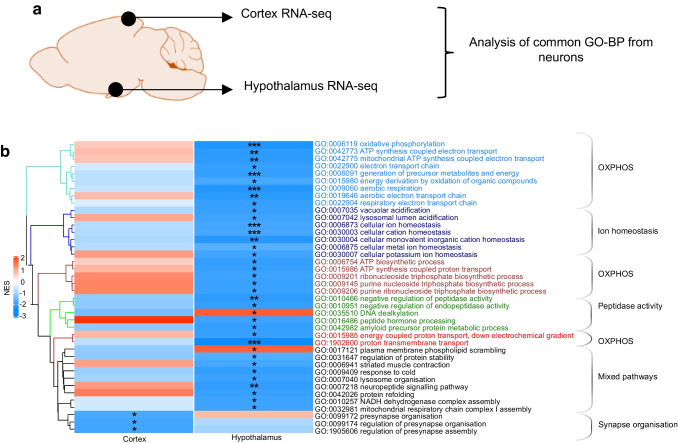
Comparison of transcriptomic analysis from the hypothalamus and cortex of HFD-/STZ-treated mice exposed to AH or RH. For the hypothalamus, samples from *n*=3 mice were pooled for each group and the experiment was repeated once to obtain a biological replicate (*n*=2 integrated datasets per condition); a dataset of 14,979 nuclei from the AH group and 14,934 nuclei from the RH group was obtained. For the cortex, snRNA-seq analysis was conducted in duplicate using nuclei from the cortex of AH- and RH-exposed mice (samples from *n*=3 mice were pooled for each group) and a total of 4650 and 9088 nuclei were obtained, respectively. (**a**) Diagram representing the strategy followed. (**b**) Hierarchical clustering of GO-BP terms enriched in neuron transcriptomes (⎸normalised enrichment score [NES]⎹ >1.7) in the cortex and hypothalamus. Corresponding *p* values are also indicated (*p<0.05, **p<0.01, ***p<0.001)

GO-BP analysis of DEGs in cortical neurons identified only a few enriched terms (ESM Fig. 9a,b, Table 4 and ESM Table 5). These were mostly related to ‘synapse organisation’ (ESM Fig. 9b, ESM Table 6) and only a few corresponded to those identified in the hypothalamus (Fig. 6a,b, and ESM Fig. 9) and often changed in the opposite direction in the two brain structures (Fig. 6b). Thus, RH induced different changes in gene expression in the hypothalamus and cortex, with more pronounced gene dysregulation in the hypothalamus.

**Table 4:** DEGs with │fold change (FC)│ >1.2 identified in neurons from the cortex of RH- vs AH-exposed mice (Bonferroni adjusted *p* value [*p*_adj_]<0.05)

Upregulated (*n*=61)	Downregulated (*n*=32)
*4921511C10Rik*	*5730522E02Rik*
*9630028H03Rik*	*Akt3*
*Agrp*	*Ankrd17*
*Arglu1*	*Brinp3*
*Armc9*	*Cnksr2*
*Atp1a1*	*Cntnap2*
*B230216N24Rik*	*Dmd*
*Brd9*	*Fgf14*
*C1ql3*	*Gm15155*
*Calm2*	*Gm38393*
*Celf4*	*Gpm6a*
*Clk1*	*Grik2*
*Cnot3*	*Gtdc1*
*Cpt1c*	*Hcn1*
*Ctnna3*	*Herc1*
*Dalrd3*	*Ilrapl1*
*Ddx5*	*Kcnj3*
*Dync1i2*	*Lingo2*
*Fgf1*	*Lrrc4c*
*Fgfr2*	*Nav3*
*Fndc9*	*Ncam2*
*Glp2r*	*Nptn*
*Gm15398*	*Nrg1*
*Gm16183*	*Nrg3*
*Gm16599*	*Nrxn1*
*Gm20642*	*Pcdh9*
*Gm29587*	*Ptprd*
*Gm34544*	*Sgcz*
*Gm35188*	*Snhg14*
*Gm36975*	*Syt14*
*Gm42439*	*Tafa2*
*Gm47423*	*Unc5d*
*Gm48678*	
*Gm49003*	
*Gsdme*	
*Hdac7*	
*Hsp90ab1*	
*Lmo4*	
*Malat1*	
*Mast3*	
*Meg3*	
*Myo18a*	
*Nisch*	
*Nme7*	
*Nmt1*	
*Nrn1*	
*P2ry14*	
*Pabpc4*	
*Pcsk2*	
*Pigk*	
*Ppia*	
*Prpf4b*	
*Ptprn*	
*Ring1*	
*Slc25a3*	
*Slc38a2*	
*Snrnp70*	
*Snx32*	
*Syt7*	
*Timm44*	
*Zcchc9*

## Discussion

**Fig. 7:**
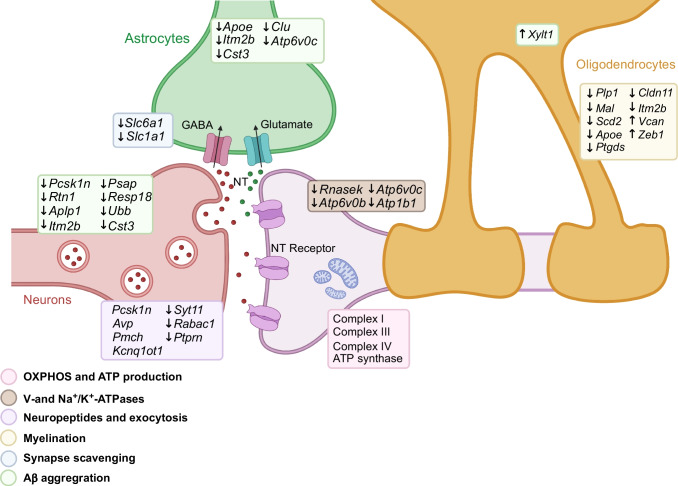
Summary of the gene expression changes that may affect tripartite synapse function. In neurons, RH reduces the expression of OXPHOS genes (pink), of V- and Na^+^/K^+^-ATPase genes (brown), and of genes controlling neuropeptide expression, processing and synaptic vesicle exocytosis (violet). A decrease in OXPHOS-related gene expression reduces ATP production and, in combination with reduced expression of the Na^+^/K^+^-ATPase genes, overactivates GI neurons. In oligodendrocytes, many genes associated with myelination were downregulated, strongly suggesting a reduction in myelin formation capacity (yellow). In astrocytes, changes in gene expression suggest reduced neurotransmitter scavenging capacity (blue). In both neurons and astrocytes, several genes were downregulated that increased propensity for Aβ formation (green). NT, neurotransmitter. Created with BioRender.com

In this study, we investigated the modifications in hypothalamic gene and protein expression induced by repeated bouts of hypoglycaemia in a mouse model of diabetes with defective glucagon secretion. Using snRNA-seq analysis, we found that RH leads to important changes in gene expression in all the cell types discussed, with sufficient information obtained from neurons, oligodendrocytes and astrocytes for reliable differential gene expression analysis. Salient findings regarding the effect of RH include the reduced expression of *Avp* and *Pmch*, two neuropeptides involved in the CRR [6, 13]. In addition, there were changes suggestive of general defects in hypoglycaemia sensing, in synaptic activity and in neuron myelination; these changes are also associated with features of neurodegenerative diseases. Proteomic analysis of hypothalamic synaptosomal fractions further indicated that defects in synaptic activity were induced by RH. These results are summarised in Fig. 7. Finally, the hypothalamus appears much more sensitive to RH than the cortex.

### Neurons

Repeated hypoglycaemia mostly induced a reduction in gene expression. Among the most downregulated genes was *Pcsk1n*, an inhibitor of the protein convertase, PCSK1. This enzyme controls the functional maturation of several hormones and neuropeptides, including AVP, and the orexigenic and anorexigenic neuropeptides of the hypothalamic melanocortin pathway. By inactivating the proconvertase, the PCKS1 inhibitor (PCSK1N) regulates neuroendocrine secretory activity and has been associated with the development of diabetes [38]. PCSK1N is also considered as a potential target for the treatment of Alzheimer’s disease due to its anti-aggregant properties [39]. Thus, downregulation of *Pcsk1n* may have an impact on various aspects of the CRR through alterations of neuropeptide production and synaptic communication.

We also observed a strong decrease in the expression of *Avp*, which encodes the precursor of AVP, a hormone produced by magnocellular neurons of the paraventricular and supraoptic nuclei of the hypothalamus [6, 40]. It is secreted in response to hypoglycaemia and stimulates glucagon secretion. Reduced AVP secretion in individuals with type 1 diabetes explains part of their defective CRR [6]. Thus, AVP-producing neurons represent an important, yet vulnerable node in the integrated neuronal circuits that control the CRR.

In neurons, a single gene was upregulated by RH, *Kcnq1ot1*, an lncRNA, the overexpression of which inhibits the expression of various genes, and which has been linked to numerous complications of diabetes [21]. Whether *Kcnq1ot1* overexpression is directly induced by hypoglycaemia and whether it contributes to the global changes in gene expression observed in our experiments will be interesting to further explore.

GO analysis of the differentially expressed neuronal genes revealed downregulation of the ‘oxidative phosphorylation’ pathway, with several genes associated with Complex I, Complex III, Complex IV and the ATP synthase being affected. These changes suggest a reduced capability to produce ATP following RH exposure. This will directly and negatively impact synaptic activity, a process that consumes a very large part of the ATP produced by mitochondria [41]. Defective mitochondrial function is also causally associated with dysregulated Ca^2+^ homeostasis and neurodegenerative diseases [42]. Several genes involved in amyloid production were also found to be dysregulated by RH, including *Aplp* [18], *Rtn1* [15] and *Itm2b* [20], all of which have been associated with amyloid beta (Aβ) protein formation, and *Resp18* [14], *Psap* [16], *Ubb* [17] and *Cst3* [19], all of which are associated with neurodegenerative diseases.

In the ‘ion homeostasis’ GO terms, the most downregulated genes include *Rnasek*, *Atp6v0b* and *Atp6v0c*, which encode subunits of the V-ATPase, and *Atp1b1*, which encodes the β1 subunit of the Na^+^/K^+^-ATPase. The V-ATPase is required for synaptic vesicle acidification and trafficking and the Na^+^/K^+^-ATPase controls membrane potential [43]; both functions are essential for sustaining normal synaptic activity. Thus, repeated episodes of decreased energy availability caused by RH induce an energy-sparing state in neurons that is suggestive of reduced ATP production and decreased expression of ATPases. Collectively, these changes induce general impairment of synaptic activity.

### Proteomic analysis of synaptosomal fractions

GO-BP analysis of proteomic data showed that the same ‘oxidative phosphorylation’ and ‘ion homeostasis’ terms that were identified in the transcriptomic analysis were downregulated by RH at the protein level. Many of the same genes and proteins associated with the OXPHOS Complex I, Complex III, Complex IV and the ATP synthase were downregulated by RH, as were those associated with the β subunit of the Na^+^/K^+^-ATPase. This indicates that a large number of the dysregulations observed at the mRNA level were reflected at the protein level. Functionally, this confirms that RH is likely to induce defective synaptic activity due to reduced OXPHOS activity and dysregulated control of membrane potential.

Among the proteins that were most differentially expressed in the synaptosomal fractions is a group of proteins involved in the control of synapse dynamics (see Fig. 5d). These included CAMK2D, a kinase activated by Ca^2+^ at excitatory synapses, which was downregulated by RH. CAMK2D activates ras-related C3 botulinum toxin substrate 1 (RAC1), a Rho-GTPase that triggers actin polymerisation, a critical process in the control of formation and function of spines and synapses [33]. This action of CAMK2D is mediated by phosphorylation of Rho-GTP exchange factors (GEFs) [34], which trigger the formation of active RAC1-GTP. Two of these Rho-GEFs, ARHGEF7 [44] and FGD5 [35], were downregulated in the RH mice and another, TIAM2, was upregulated. In contrast, among the proteins overexpressed after RH exposure are three Rho-GTPase-activating proteins (Rho-GAPs), CTNND2, ARHGAP25 and USP6NL, which increase GTP hydrolysis and RAC1 inactivation. These observations indicate important changes leading to reduced RAC1 activity. Interestingly, there was also decreased expression of ADAM22, a receptor for leucine-rich glioma-inactivated protein 1 beta (LGI1ß), which regulates synaptic maturation and activity [45]. Together, these observations indicate that RH leads to reduced formation of spines and synapses.

### Oligodendrocytes

In oligodendrocytes, the major changes in gene expression induced by RH were related to ‘lipid biosynthesis’ and ‘myelination’, with decreased expression of the following: *Plp1*, the major constituent of myelin; *Mal*, which is associated with myelin formation; *Scd2*, which is the main stearoyl-CoA desaturase (SCD) isoform in the brain, the dysregulation of which is associated with impaired myelin formation and neurodegenerative diseases [46]; *Apoe*, implicated in brain lipid metabolism and neurodegeneration [28]; *Ptgds* which catalyses the formation of prostaglandin D2 (PGD2), a promoter of oligodendrocyte development and myelination [47]; and *Cldn11*, which is specifically required for the formation of myelin-associated tight junctions and the absence of which leads to major defects in neuron myelination [48]. Among the few upregulated mRNAs in oligodendrocytes from RH-exposed mice were *Xylt1*, required for heparan sulfate proteoglycan (HSPG) biosynthesis. HSPGs increase uptake of monomeric Tau protein, which induces an inflammatory response in neurons and astrocytes [49] and also blocks axon growth [50]. The other upregulated genes were *Vcan*, encoding a chondroitin sulfate proteoglycan that inhibits myelination of oligodendrocyte precursors [51], and *Zeb1*, a zinc finger transcription factor that induces neuroinflammation [52]. Together, these changes in oligodendrocyte gene expression indicate a generalised defect in myelin formation and the induction of local inflammation.

### Astrocytes

In astrocytes, the most downregulated genes were *Apoe, Itm2b*, *Itm2c*, *Cst3*, *Clu* and *Atp6v0c*. Both *Itm2b* and *Itm2c* (a paralogue of *Itm2b*) are negative regulators of Aβ production, and *Cst3* and *Clu* prevent aggregation of amyloid fibrils. Together, *Itm2b*, *Itm2c*, *Cst3* and *Clu* potentially ameliorate Alzheimer’s disease [29, 53]. In addition, *Atp6v0c* encodes the pore-forming subunit of the V-ATPase, which is required to acidify lysosomes in which Aβ fibrils can be degraded [54].

The downregulated ‘ion transport’ GO-BP term is enriched in genes that encode voltage-gated K^+^ channels (*Kcnj10*, *Kcnj2*, *Kcnh8*), GABA receptors (*Gabrg3*, *Gabbr1*), the GABA transporter *Slc6a1*, and the glutamate transporter *Slc1a1*. These genes are required in astrocytes to clear neuronal synapses from excess neurotransmitters [55]. These changes in gene expression further suggest a generalised defect in tripartite synaptic activity and increased tendency to form Aβ peptides.

### Cortex

Cortical neurons also displayed marked changes in gene expression in RH- vs AH-exposed mice. These were related mainly to presynapse organisation, with dysregulation in the synaptic regulators *Nlgn1*, *Magi2*, *Il1rapl1*, *Nrxn1*, *Slitrk1*, *Lrfn5* and *Mdga2* [56, 57]. However, the dysregulated genes observed in the cortex were very different than those observed in the hypothalamus of the same mice. Together, these findings indicate that the cortex is less and differently sensitive to RH than the hypothalamus.

### Defective counterregulation

Defective counterregulation involves not only impaired glucagon secretion but also reduced secretion of adrenaline, noradrenaline, cortisol and growth hormones. These defects are thought to be caused by the progressive loss of central hypoglycaemia sensing and reduced activation of the autonomic nervous system, the HPA axis and AVP secretion. Mechanistically, activation of GI neurons by hypoglycaemia is triggered by a fall in intracellular ATP levels, which reduces the activity of the Na^+^/K^+^-ATPase, leading to membrane depolarisation and neuron firing [10]. For instance, a specific role of this signalling pathway in linking the activation of arcuate nucleus agouti-related peptide (AgRP) GI neurons to vagal nerve activity and glucagon secretion has been recently reported [58]. Thus, the observed decrease in neuronal expression of OXPHOS and Na^+^/K^+^-ATPase genes suggests over-activation of GI neurons in RH-exposed mice, which may also lead to their desensitisation to subsequent hypoglycaemic challenges. In addition, we observed decreased expression of *Avp* and of *Pmch*, which control glucagon [7] and corticosterone [13] secretion, respectively. Collectively, these changes in gene expression may not only explain basal hyperglucagonaemia in RH-exposed mice, but also the impaired response to subsequent hypoglycaemic episodes in animals with defective counterregulation.

### Neurodegenerative diseases

Our data showed that several hypothalamic gene expression changes in RH- vs AH-exposed mice involved processes associated with neurodegenerative diseases, in particular Alzheimer’s disease, which is characterised by defects in energy homeostasis, synaptic and neuronal network dysfunction, and increased propensity for Aβ production (see Fig. 7) [59]. It is worth noting that snRNA-seq data showed that different modifications in gene expression was induced by RH in the cortex of the same mice. These were mostly related to synaptic activity and less so related to neurodegenerative pathways. These findings further indicate the specific, high sensitivity of the hypothalamus to the metabolic challenges imposed by RH.

### Limitations of the study

The mouse model used has several limitations. First, our model of type 2 diabetes, with a degree of insulinopaenia, is more characteristic of long-term type 2 diabetes and does not fully represent the human condition. Second, our analysis did not identify whether the observed changes were specific to selected hypothalamic nuclei, or to GI neurons or neurons activated by high glucose (glucose-excited [GE] neurons). Also, our snRNA-seq analysis did not yield sufficient information on all hypothalamic cell types, therefore impairing the detection of potential relevant dysregulation in other cell types. Finally, whether the modifications in gene expression and glucagon secretion are reversible upon extended therapeutic restoration of normoglycaemia is not known.

In summary, we show that RH has an impact on all major hypothalamic cell types (Fig. 7). In neurons, it decreases the expression of OXPHOS genes and of plasma membrane and vacuolar ATPases that regulate synaptic activity, whilst in oligodendrocytes, RH downregulates the expression of genes controlling myelin formation and, in astrocytes, several genes controlling neurotransmitters scavenging at the synapse. In addition, many of the changes observed in neurons, oligodendrocytes and astrocytes suggest increased propensity for Aβ formation and accumulation. Importantly, defective CRR could be explained by the specific downregulation of *Pcsk1*, *Avp* and *Pmch,* and by the decreased expression of OXPHOS and Na^+^/K^+^-ATPase genes that may lead, not only to over-activation of hypoglycaemia-sensing neurons and increased basal hyperglucagonaemia, but also to impaired activation of these neurons during subsequent hypoglycaemic episodes. Together, our findings illustrate the very high sensitivity of the hypothalamus and its constituent cells to RH. They provide a framework to design novel experimental approaches to test the role of the identified pathways in defective counterregulation.

### Supplementary Information

Below is the link to the electronic supplementary material.

Supplementary file1 (PDF 6311 KB)

Supplementary file2 (XLSX 5288 KB)

## Data Availability

All transcriptomic data are available via the GEO (http://www.ncbi.nlm.nih.gov/geo/), using the accession no. GSE226277. Proteomic data are available via the ProteomeXchange data repository (http://www.proteomexchange.org), using the accession no. PXD040183.

## Abbreviations

Aβ: Amyloid beta
ADAM22: Disintegrin and metalloproteinase domain-containing protein 22
AH: Acute hypoglycaemia
ARHGAP25: Rho GTPase-activating protein 25
ARHGEF7: Rho guanine nucleotide exchange factor 7
AVP: Arginine vasopressin
CAMK2D: calcium/calmodulin dependent protein kinase II delta
CRR: Counterregulatory response
CTNND2: Catenin delta-2
DEG: Differentially expressed gene
FGD5: FYVE, RhoGEF and PH domain-containing protein 5
GABA: γ-Aminobutyric acid
GEF: GTP exchange factor
GI: Glucose-inhibited (neurons)
GO: Gene ontology
GO-BP: Gene ontology biological process
GSEA: Gene-set enrichment analysis
HFD: High-fat diet
HPA: Hypothalamic–pituitary–adrenal
HSPG: Heparan sulfate proteoglycan
KEGG: Kyoto Encyclopedia of Genes and Genomes
lncRNA: Long non-coding RNA
OXPHOS: Oxidative phosphorylation
PCSK1: Proprotein convertase subtilisin/kexin type 1
PCKS1N: Proprotein convertase subtilisin/kexin type 1 inhibitor
QC: Quality control
RAC1: Ras-related C3 botulinum toxin substrate 1
RH: Recurrent hypoglycaemia
snRNA-seq: Single-nuclei RNA-seq
STZ: Streptozocin
TIAM2: T cell lymphoma invasion and metastasis 2
USP6NL: USP6 N-terminal-like protein
V-ATPase: Vacuolar H^+^-ATPase
