# Correction to: Disrupted hypothalamic transcriptomics and proteomics in a mouse model of type 2 diabetes exposed to recurrent hypoglycaemia

**DOI:** 10.1007/s00125-023-06083-3

**Published:** 2024-01-11

**Authors:** Judit Castillo‑Armengol, Flavia Marzetta, Ana Rodriguez Sanchez-Archidona, Christian Fledelius, Mark Evans, Alison McNeilly, Rory J. McCrimmon, Mark Ibberson, Bernard Thorens

**Affiliations:** 1 Novo Nordisk A/S, Malov, Denmark; 2 Center for Integrative Genomics (CIG), University of Lausanne, Lausanne, Switzerland; 3 Vital‑IT Group, SIB Swiss Institute of Bioinformatics, Lausanne, Switzerland; 4 IMS Metabolic Research Laboratories, Addenbrookes Biomedical Campus, Cambridge, UK; 5 School of Medicine, University of Dundee, Dundee, UK

**Correction to: Diabetologia**

10.1007/s00125-023-06043-x

The affiliations for Mark Evans, Alison McNeilly and Rory J. McCrimmon have been corrected.

The original article has been corrected.

