# Supplementary material for: Disrupted hypothalamic transcriptomics and proteomics in a mouse model of type 2 diabetes exposed to recurrent hypoglycaemia
Source: Diabetologia. 2023 Nov 28;67(2):371–91. doi: 10.1007/s00125-023-06043-x (PMC10789691; doi:10.1007/s00125-023-06043-x)
Supplement: Supplementary file 1 — Supplementary file1 (PDF 6311 KB) [file 125_2023_6043_MOESM1_ESM.pdf]

ESM Methods

Animals. 7-weeks old C57BL6N male mice were purchased from Charles River Laboratories (Saint Germain Nuelles, France). Animals were housed on a 12-h light/dark cycle with free access to food and water. All animal experimentations were approved by the Veterinary Office of Canton de Vaud under license agreement VD 3535.

High-fat diet and streptozocin treatment of mice. Mice were fed a high fat diet (HFD; 15.6% proteins, 44.4% lipids, 40.3% carbohydrates; Diet 235HF, Safe, Paris, France) and seven weeks after initiation of the diet they received intraperitoneal (i.p.) injections, after a 5h fasting period, of streptozocin (S0130, Sigma Aldrich, St Louis, MO, USA) dissolved in citrate buffer pH 4.5, two times 35mg/kg and once 70mg/kg, the injections being performed very other days. One week after the last injection, glycaemia was measured using a glucometer (Contour XT, Bayer, Leverkursen, Germany).

Insulin-induced hypoglycaemia and glucagon measurements. T2D mice were divided in two groups with similar body weight and glycaemia. Only animals with basal glycaemia>9.5mmol/l after the streptozocin were considered for the experiment. To induce hypoglycaemia, animals were placed in individual buckets without access to food. One group of mice (Recurrent Hypoglycaemia, RH) received three insulin (Actrapid, Novo Nordisk, Bagsvaerd, Denmark) i.p. injections per week during four weeks (12 injections). The insulin dose injected was adjusted according to the basal glycaemia of the mice and ranged from 2-7U/kg. The second group of mice received three i.p. injections per week of saline (Acute Hypoglycaemia, AH) and a final injection of insulin. Tail blood glucose concentrations were monitored at times -60min, 60min and 120min after the IP injection using a glucometer (Contour XT, Bayer, Leverkursen, Germany). One hour after the last i.p. injection of insulin, mice were anesthetized using isoflurane and blood was collected from the submandibular vein and plasma glucagon levels were using the Mercodia glucagon ELISA (10-1271-01, Mercodia, Uppsala, Sweden). Brains were collected and snap frozen 120 min after the last insulin IP injection.

Nuclei isolation. Hypothalami (~Bregma -0.10mm to -1.9 mm) and cortex samples (~Bregma +1.10mm to -0.10mm including the motor, somatosensory and cingulate cortex) were dissected, rinsed in cold PBS, and nuclei were isolated following a modified version of the

protocol CG000375 recommended by 10x Genomics (Pleasanton, California, USA). Tissue was homogenized using a motorized pestle mixer and incubated in lysis buffer (10mM Tris-HCl pH7.4 (T2194, Sigma Aldrich, St Louis, MO, USA), 10mM NaCl (59222C, Sigma Aldrich, St Louis, MO, USA), 3mM $MgCl_2$ (M1028, Sigma Aldrich, St Louis, MO, USA), 1mM DTT (646563, Sigma Aldrich, St Louis, MO, USA), 0.1% Nonidet P 40 (74385, Sigma Aldrich, St Louis, MO, USA), 1U/μL RNase inhibitor (3335402001, Roche, Basel, Switzerland)). Then, the lysates were filtered through a 70μm strainer and spun at 500g for 5min at 4°C. Pellets were washed with 1%BSA-PBS and filtered with a 40μm cell strainer. The flow-through was spun at 500g for 5min at 4°C and pellets were resuspended in 1%BSA-PBS with 7-AAD (7240-37-1, Sigma Aldrich, St Louis, MO, USA). Nuclei were sorted by gating 7-AAD+ nuclei on a FACS Aria II Instrument using a 100μm nozzle. Sorted nuclei were permeabilized in 10mM Tris-HCl pH7.4 (T2194, Sigma Aldrich, St Louis, MO, USA), 10mM NaCl (59222C, Sigma Aldrich, St Louis, MO, USA), 3mM $MgCl_2$ (M1028, Sigma Aldrich, St Louis, MO, USA), 1% BSA (130-091-376, Mylteni Biotech, Bergisch Gladbach, Germany), 1mM DTT (646563, Sigma Aldrich, St Louis, MO, USA), 0.01% Nonidet P 40 (74385, Sigma Aldrich, St Louis, MO, USA), 0.01% Tween 20 (A4974.0500, Panreac Applichem, Darmstadt, Germany), 0.001% Digitonin (BN2006, Thermofisher, Waltham, Massachusetts, USA), 1U/μL RNase inhibitor (3335402001, Roche, Basel, Switzerland)), washed in 1%BSA-PBS and spun at 500g for 5min at 4°C. Nuclei were resuspended in nuclei buffer (10x Genomics, Pleasanton, California, USA) with 1mM DTT and 1U/μL RNase inhibitor and counted using the Moxi V counter (MXV102, Orflo Technologies, Ketchum, Idaho, USA).

Single-nuclei RNA sequencing. Hypothalamic single nuclei libraries were prepared using 10x Genomics Chromium single cell multiome ATAC + gene expression assay. Cortical single nuclei libraries were prepared using Chromium single cell 3' RNA-seq protocol. All libraries were sequenced on Illumina NovaSeq 6000. For multiome samples, the resulting FASTQ files were processed with the cellranger-arc count (v2.0.0, 10x Genomics) from the Cell Ranger Multiome pipeline with the default settings on the Mus musculus reference (refdata-cellranger-arc-mm10-2020-A-2.0.0). 3RNA samples were processed with the cellranger count (v6.0.0, 10x Genomics) from the Single Cell Gene Expression pipeline on the Mus musculus reference (refdata-gex-mm10-2020-A), specifying the expected number of cells equal to 10'000 and retaining intron mapping reads in the analysis. 20’000 nuclei were incubated in a transposition mix that fragments the DNA in open regions of the chromatin. Then the nuclei

were partitioned in a Chromium Next GEM chip J. The sequencing libraries were prepared with the Chromium Single Cell multiome ATAC + Gene Expression reagents strictly following the manufacturer's recommendations. Sequencing of RNAseq libraries was performed on the Illumina HiSeq 4000 using HiSeq 3000/4000 SBS Kit reagents according to 10X Genomics recommendations (28 cycles read1, 10 cycles i7 and i5 index reads and 90 cycles read2).
The sequencing data were demultiplexed with the bcl2fastq Conversion Software (v. 2.20, Illumina). For multiome samples, the resulting FASTQ files were processed with the cellranger-arc count (v2.0.0, 10x Genomics) from the Cell Ranger Multiome pipeline with the default settings on the Mus musculus reference (refdata-cellranger-arc-mm10-2020-A-2.0.0). 3RNA samples were processed with the cellranger count (v6.0.0, 10x Genomics) from the Single Cell Gene Expression pipeline on the Mus musculus reference (refdata-gex-mm10-2020-A), specifying the expected number of cells equal to 10'000 and retaining intron mapping reads in the analysis.

Single-nuclei sequence analysis. The R package Seurat (v4.1.1) [1] was used for downstream analysis. Gene expression data from hypothalamic single-nucleus multiome samples were extracted and analysed independently from the ATAC-seq assays. To filter out possible doublets and low-quality cells, we removed nuclei with ≤ 300 or ≥7500 detected genes and >20% mitochondrial genes. Gene expression counts for each nucleus were normalized by the total expression, multiplied by a scaling factor of 10,000 followed by log-transformation. We identified anchors between datasets using the FindIntegrationAnchors function with the top 2000 most variable genes as input and performed data integration with reciprocal principal component analysis (rPCA) using the IntegrateData function. After scaling and centering the integrated dataset, we performed dimensional reduction with PCA followed by uniform manifold approximation and projection (UMAP) using the significant top 40 principal components estimated using the JackStrawPlot function. Unsupervised clustering of nuclei was carried out by constructing a shared nearest neighbor (SNN) graph according to K-nearest neighbors (K = 20) and then determining the number of clusters with a modularity function optimizer using the functions FindNeighbors and FindClusters using 0.025 as resolution parameter. In total, 31 clusters were identified.
Cortex RNA-seq data were processed using the same pipeline. A total of 19 clusters were identified which were annotated as specific cell types using the same list of tissue-specific markers. To assign cell-type identity to each cluster we evaluated the expression profile of a

custom list of markers (neurons: Snap25, Syt1; oligodendrocytes: Mobp, Pdgfra, Apod; astrocytes: Gja1, Agt, Slc1a3; microglia: C1qa, Cx3cr1, P2ry12; endothelial: Cldn5; pericytes: Abcc9), which enable the identification of the major brain cell types, namely neurons, astrocytes, oligodendrocytes and microglia, as well as additional cell types such as pericytes and endothelial cells. The population of neurons was also divided between GABAergic and Glutamatergic subtypes according to the expression score of the GABAergic markers GAD1 and GAD2 and the Glutamatergic marker Slc17a6. To identified differentially expressed genes between recurrent and acute hypoglycaemic samples for each cell type, we used FindMarkers function with Wilcoxon rank-sum test, “min.pct=0.01” and “logfc.threshold=0” parameters and Bonferroni p-value adjustment. We considered, as differentially expressed, those genes with padj<0.05 and greater than 1.2 fold-change.

Gene set enrichment analysis. The Gene Ontology Biological Pathway (GOBP) and Kyoto Encyclopedia of Genes and Genomes (KEGG) terms enrichment analysis was performed by a gene-set enrichment analysis (GSEA) approach using the R package ClusterProfiler (v4.4.4) [2] on the entire lists of expressed genes preranked by signed P-value as determined by Wilcoxon rank-sum test. A Benjamini-Hochberg correction was applied to correct for multiple comparisons. Unless otherwise stated, an enrichment was deemed significant if padj < 0.05. Enriched gene sets were visualized as enrichment maps using the emapplot function. Enrichment maps organize enriched gene sets in a network where edges connect terms that meet a certain threshold of similarity derived by Jaccard's correlation coefficient. In order to compare the perturbation of functional profiles in neurons, enriched GOBP terms in hypothalamus or cortex were hierarchically clustered according to their semantic similarity based on the graph structure of GO (Wang measure) using the R package GOSemSim (v2.22.0) [3]. Results were then plotted as a heatmap with the R package ComplexHeatmap (v2.12.1) [4].

Synaptosome preparation. Hypothalamus samples were homogenized using a motorized pestle mixer in hypotonic cell lysis buffer (HBLC) containing 20mM HEPES (H0887, Sigma Aldrich, St Louis, MO, USA), 10mM NaCl (59222C, Sigma Aldrich, St Louis, MO, USA), 1.5mM $MgCl_2$ (M1028, Sigma Aldrich, St Louis, MO, USA), 0.2mM EDTA (A4892.1000, Panreac Applichem, Darmstadt, Germany), 0.1% Nonidet P 40 (74385, Sigma Aldrich, St Louis, MO, USA) and 20% Glycerol (G5516, Sigma Aldrich, St Louis, MO, USA) and incubated on ice for

45min. The homogenate was spun at 1000 x g for 10min at 4°C. The supernatant was collected and this step was repeated to remove the remaining nuclear fraction. The resulting supernatant was spun at 12000 x g for 10min at 4°C and the pellet containing the synaptosomal fraction was resuspended in HBLC. Protein was quantified using BCA (23225, Thermofisher, Waltham, Massachusetts, USA).

Proteomics sample preparation. The synaptosomal fractions of the hypothalami from 4 AH and 4 RH mice were digested following the SP3 method [5] using magnetic Sera-Mag Speedbeads (Cytiva 45152105050250, 50 mg/ml). Briefly, synaptosomal fractions samples (in 15μl HCLB buffer) were used for digestion. After 10 min heating at 75°C, proteins were alkylated with 30mM chloroacetamide for 45 min at RT in the dark. Beads were added at a ratio 10:1 (w:w) to samples, and proteins were precipitated on beads with ethanol (final concentration: 60 %). After 3 washes with 80% ethanol, beads were digested in 50μl of 100 mM ammonium bicarbonate with 1.2 μg of trypsin. After 1h of incubation at 37°C, the same amount of trypsin was added to the samples for an additional 1h of incubation. Supernatant were then recovered, transferred to new tubes and acidified with formic acid (0.5% final concentration). Aliquots (1/8) of samples were mixed to create a pool. Then, this was separated into 6 fractions by off-line basic reversed-phase (bRP) using the Pierce High pH Reversed-Phase Peptide Fractionation Kit (Thermo Fisher Scientific, Waltham, Massachusetts, USA). The fractions were collected in 7.5, 10, 12.5, 15, 17.5 and 50% acetonitrile in 0.1 % triethylamine (~pH 10). Dried bRP fractions were redissolved in 50μl 2% acetonitrile with 0.5% TFA, and 3-5μl were injected for LC-MS/MS analyses.

LC-MS analyses. LC-MS/MS analyses were carried out on a TIMS-TOF Pro (Bruker, Bremen, Germany) mass spectrometer interfaced through a nanospray ion source ("captive spray") to an Ultimate 3000 RSLCnano HPLC system (Dionex). Peptides were separated on a reversed-phase custom packed 40 cm C18 column (75 μm ID, 100Å, Reprosil Pur 1.9 um particles, Dr. Maisch, Germany) at a flow rate of 0.250 μl/min with a 2-27% acetonitrile gradient in 93 min followed by a ramp to 45% in 15 min and to 90% in 5 min (all solvents contained 0.1% formic acid). Identical LC gradients were used for DDA and DIA measurements.

For creation of the spectral library, data-dependent acquisitions (DDA) were carried out on the 6 bRP fractions of both pools using a standard TIMS PASEF method [6] with ion accumulation for 100 ms for each survey MS1 scan and the TIMS-coupled MS2 scans. Duty

cycle was kept at 100%. Up to 10 precursors were targeted per TIMS scan. Precursor isolation was done with a 2 Th or 3 Th windows below or above m/z 800, respectively. The minimum threshold intensity for precursor selection was 2500. If the inclusion list allowed it, precursors were targeted more than one time to reach a minimum target total intensity of 20'000. Collision energy was ramped linearly based uniquely on the 1/k0 values from 20 (at 1/k0=0.6) to 59 eV (at 1/k0=1.6). Total duration of a scan cycle including one survey and 10 MS2 TIMS scans was 1.16 s. Precursors could be targeted again in subsequent cycles if their signal increased by a factor 4.0 or more. After selection in one cycle, precursors were excluded from further selection for 60s. Mass resolution in all MS measurements was approximately 35'000. The data-independent acquisition (DIA) used mostly the same instrument parameters as the DDA method and was as reported previously [7]. Per cycle, the mass range 400-1200 m/z was covered by a total of 32 windows, each 25 Th wide and a 1/k0 range of 0.3. Collision energy and resolution settings were the same as in the DDA method. Two windows were acquired per TIMS scan (100ms) so that the total cycle time was 1.7 s.

Proteomics data analysis. Raw Bruker MS data were processed directly with Spectronaut 15.7 (Biognosys, Schlieren, Switzerland). A library was constructed from the DDA bRP fraction data by searching the reference mouse proteome (www.uniprot.org) database of June 4th, 2021 (55'364 sequences). For identification, peptides of 7-52 AA length were considered, cleaved with trypsin/P specificity and a maximum of 2 missed cleavages. Carbamidomethylation of cysteine (fixed), methionine oxidation and N-terminal protein acetylation (variable) were the modifications applied. Mass calibration was dynamic and based on a first database search. The Pulsar engine was used for peptide identification. Protein inference was performed with the IDPicker algorithm. Spectra, peptide and protein identifications were all filtered at 1% FDR against a decoy database. Specific filtering for library construction removed fragments corresponding to less than 3 AA and fragments outside the 300-1800 m/z range. Also, only fragments with a minimum base peak intensity of 5% were kept. Precursors with less than 3 fragments were also eliminated and only the best 6 fragments were kept per precursor. No filtering was done on the basis of charge state and a maximum of 2 missed cleavages was allowed. Shared (non proteotypic) peptides were kept. The library created contained 104,951 precursors mapping to 81,168 stripped sequences, of which 33,796 were proteotypic. These corresponded to 8,292 protein groups (11,697 proteins). Of these, 1,217 were single hits (one peptide precursor). In total 619,559 fragments were used for quantitation. Peptide-centric

analysis of DIA data was done with Spectronaut 15.7 using the library described above. Single hits proteins (defined as matched by one stripped sequence only) were kept in the Spectronaut analysis. Peptide quantitation was based on XIC area, for which a minimum of 1 and a maximum of 3 (the 3 best) precursors were considered for each peptide, from which the median value was selected. Quantities for protein groups were derived from inter-run peptide ratios based on MaxLFQ algorithm [8]. Global normalization of runs/samples was done based on the median of peptides.

Bioinformatic analysis from proteomics data. Statistical analyses of proteomics data were done with the Perseus software package (version 1.6.15.0) [9]. Contaminant proteins were removed, and intensity values were log2-transformed. After assignment to groups, only proteins quantified in at least 4 samples of one group were kept. After missing values imputation (based on normal distribution using Perseus default parameters), t-tests were carried out among all conditions, with permutation-based FDR correction for multiple testing (Q-value threshold <0.05). Proteins were preranked by signed t-test P-value and subjected to GSEA using GOBP ontologies, as previously described. GOBP terms commonly enriched in proteomics data and hypothalamic neuron gene profiles were hierarchically clustered and plotted as a heatmap.

Statistical analysis. Unless stated otherwise, data are expressed as mean ± SEM. Statistical analysis was performed using GraphPad Prism 8.4.0 (Graphpad Software), either by a mixed-effects analysis followed by a Sidak's post hoc test, a repeated-measures two-way ANOVA followed by a Sidak's post hoc test, or by an unpaired two-tailed Student's t-test. P-values from <0.05 were considered to be significant. Bonferroni and Benjamini-Hochberg corrections for were applied for multiple comparisons.

References

[1] Butler A, Hoffman P, Smibert P, Papalexi E, Satija R (2018) Integrating single-cell transcriptomic data across different conditions, technologies, and species. Nat Biotechnol 36(5): 411-420

[2] Wu T, Hu E, Xu S, et al. (2021) clusterProfiler 4.0: A universal enrichment tool for interpreting omics data. Innovation (Camb) 2(3): 100141

[3] Yu G (2020) Gene Ontology Semantic Similarity Analysis Using GOSemSim. Methods Mol Biol 2117: 207-215

[4] Gu Z, Eils R, Schlesner M (2016) Complex heatmaps reveal patterns and correlations in multidimensional genomic data. Bioinformatics 32(18): 2847-2849

[5] Hughes CS, Moggridge S, Muller T, Sorensen PH, Morin GB, Krijgsveld J (2019) Single-pot, solid-phase-enhanced sample preparation for proteomics experiments. Nat Protoc 14(1): 68-85. 10.1038/s41596-018-0082-x

[6] Meier F, Brunner AD, Koch S, et al. (2018) Online Parallel Accumulation-Serial Fragmentation (PASEF) with a Novel Trapped Ion Mobility Mass Spectrometer. Mol Cell Proteomics 17(12): 2534-2545. 10.1074/mcp.TIR118.000900

[7] Meier F, Brunner AD, Frank M, et al. (2020) diaPASEF: parallel accumulation-serial fragmentation combined with data-independent acquisition. Nat Methods 17(12): 1229-1236. 10.1038/s41592-020-00998-0

[8] Cox J, Hein MY, Luber CA, Paron I, Nagaraj N, Mann M (2014) Accurate proteome-wide label-free quantification by delayed normalization and maximal peptide ratio extraction, termed MaxLFQ. Mol Cell Proteomics 13(9): 2513-2526

[9] Tyanova S, Temu T, Sinitcyn P, et al. (2016) The Perseus computational platform for comprehensive analysis of (prote)omics data. Nature methods 13(9): 731-740

ESM Figures

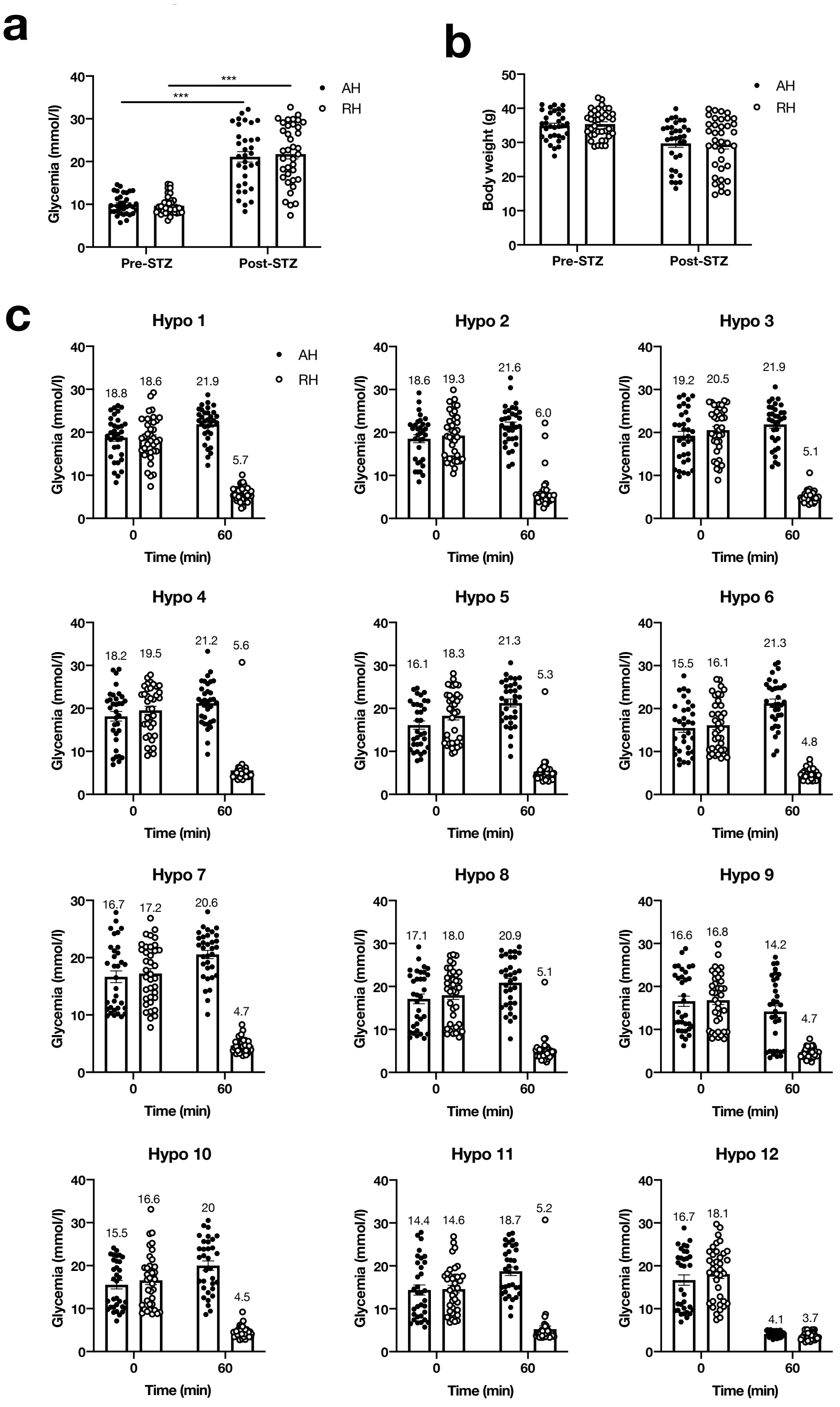

ESM Fig. 1. Generation of a model of HFD/STZ exposed to AH or RH. (a) Glycaemia (mmol/l) and (b) Body weight (g) of C57BL/6N-HFD mice before and after STZ injection for the AH and RH groups (c) Glycaemia before and 60 minutes after the 12 rounds of saline or insulin injections in the AH and RH groups (AH: n=33, RH: n=37). Data are means ± SEM. ***p<0.001.

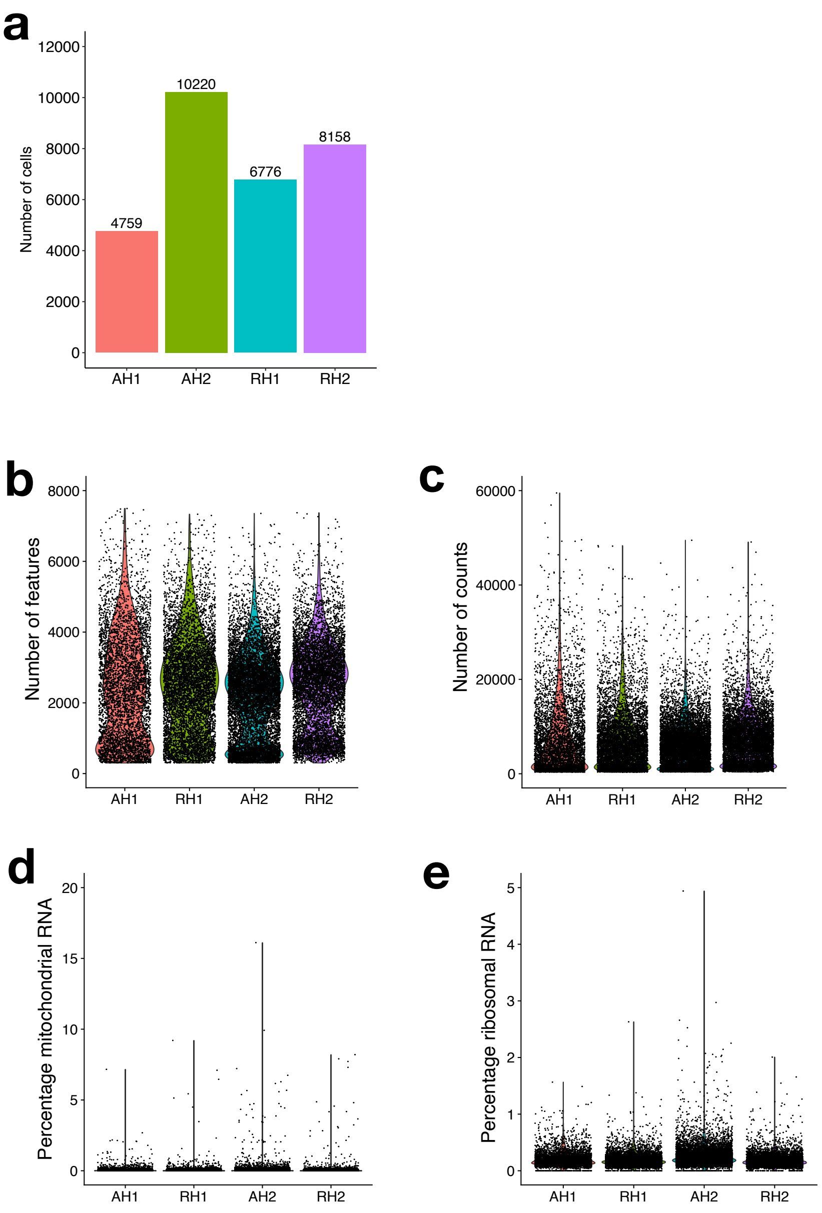

ESM Fig. 2. Post-filtering quality control metrics of hypothalamus nuclei. (a) Number of nuclei isolated from the hypothalamus of type 2 diabetic mice exposed to AH (AH1, AH2) or RH (RH1, RH2). Violin plots illustrating per-cell QC metrics: (b) nFeature (number of genes with at least one UMI count); (c) nCount (number of UMI counts); (d) percentage of mitochondrial genes; (e) percentage of ribosomal genes.

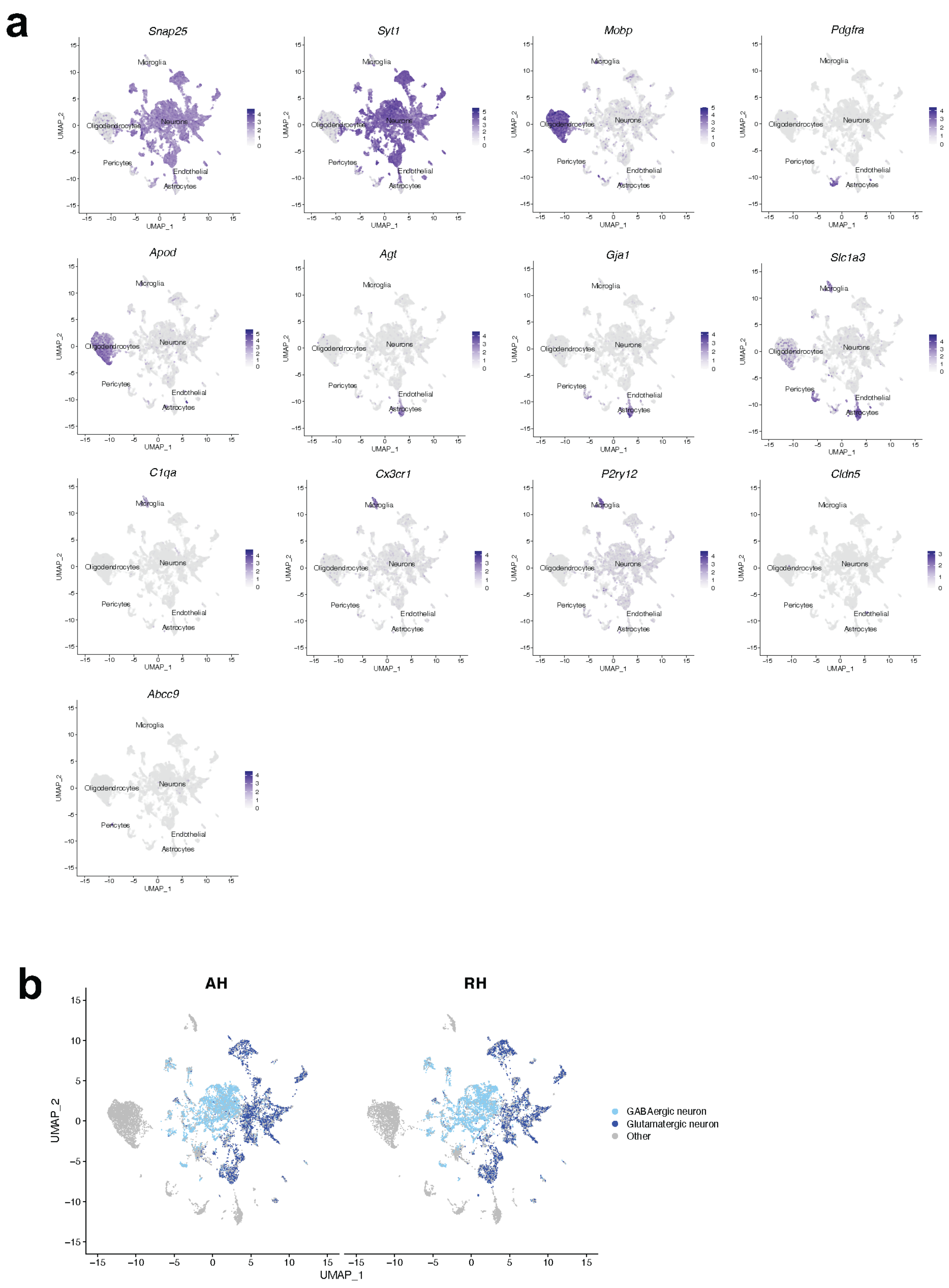

ESM Fig. 3. Annotation of hypothalamic nuclei. (a) UMAP plots showing the expression of canonical markers for the six cell categories (neurons, oligodendrocytes, astrocytes, microglia, endothelial cells and pericytes). (b) UMAP plot showing the GABAergic and Glutamatergic neurons in AH and RH mice.

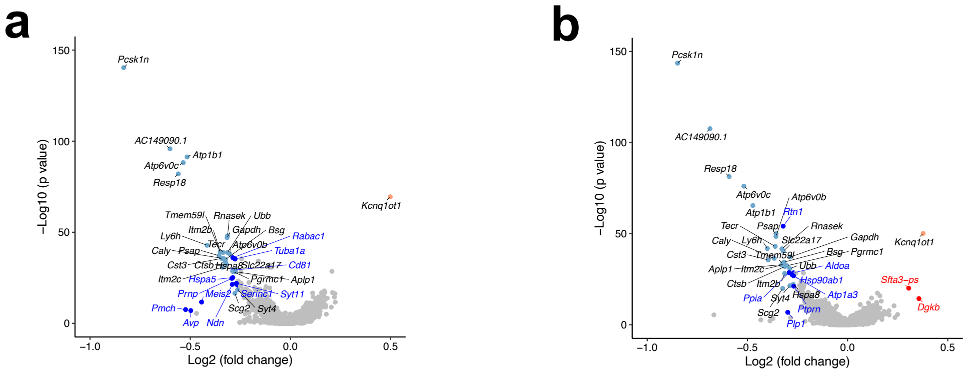

ESM Fig. 4. Transcriptional analysis of GABAergic and Glutamatergic neurons from the cortex of HFD/STZ mice exposed to AH or RH. (a) Volcano Plot depicting genes expressed in hypothalamic GABAergic or (b) glutamatergic neurons from mice subjected to RH as compared to AH. Red dots depict upregulated genes and blue dots depict downregulated genes (fold-change >1.2 or <-1.2 and Bonferroni adjusted P value < 0.05). DEGs specific to the neuron type are indicated with the font in colour and common DEGs are labelled with black font.

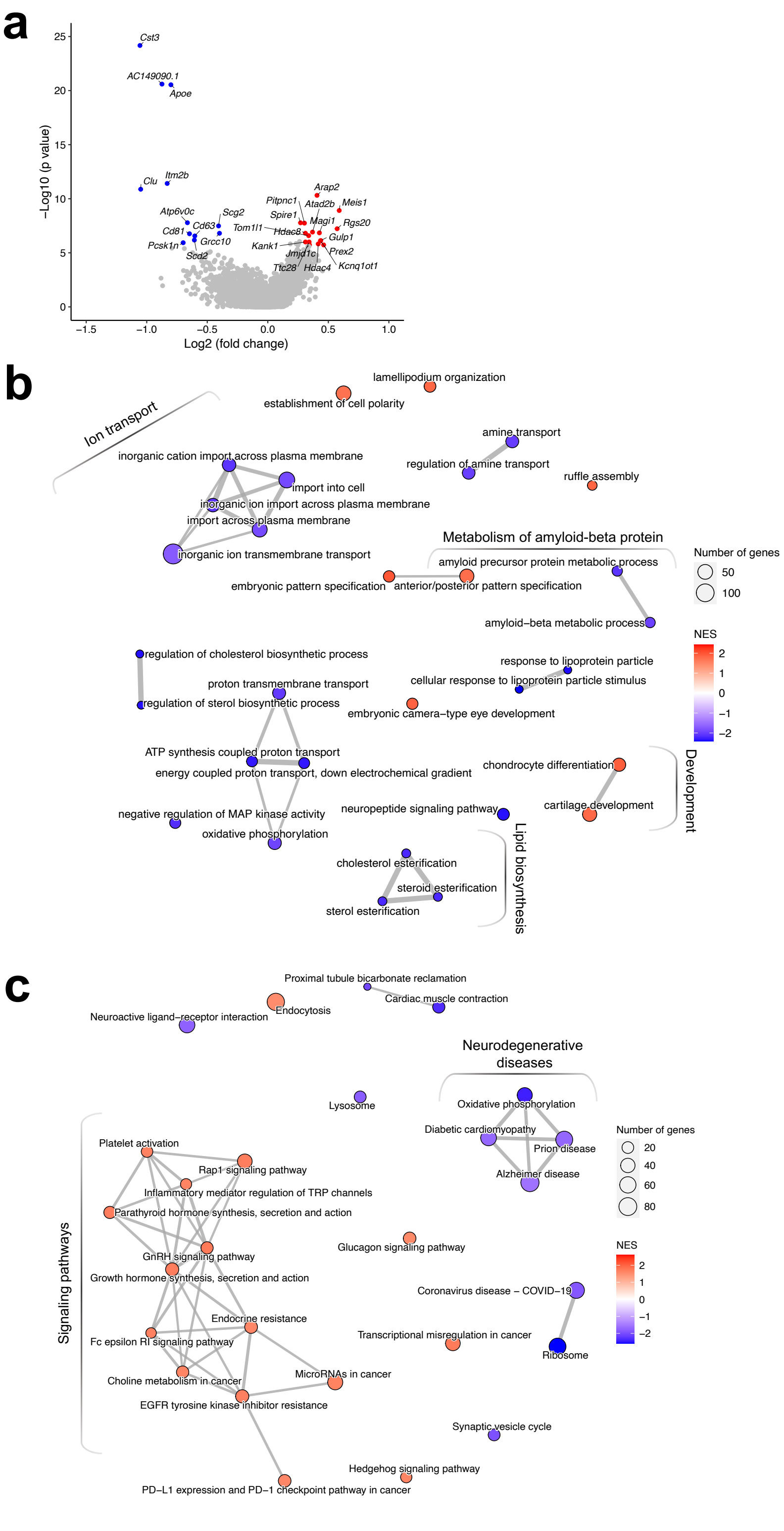

ESM Fig. 5. Transcriptional analysis of astrocytes from hypothalamus of HFD/STZ mice exposed to AH or RH. (a) Volcano Plot depicting genes expressed in hypothalamic astrocytes from mice subjected to RH as compared to AH. Red dots depict upregulated genes and blue dots depict downregulated genes (fold-change >1.2 or <-1.2 and Bonferroni adjusted P value < 0.05). (b) Network visualization of the top enriched GO-BP or (c) KEGG terms in DE genes between RH vs AH mice. The node colour indicates the normalized enrichment score (NES), the node size indicates the number of core-enriched genes overlapping gene count and the edges represent the pairwise similarity between terms.

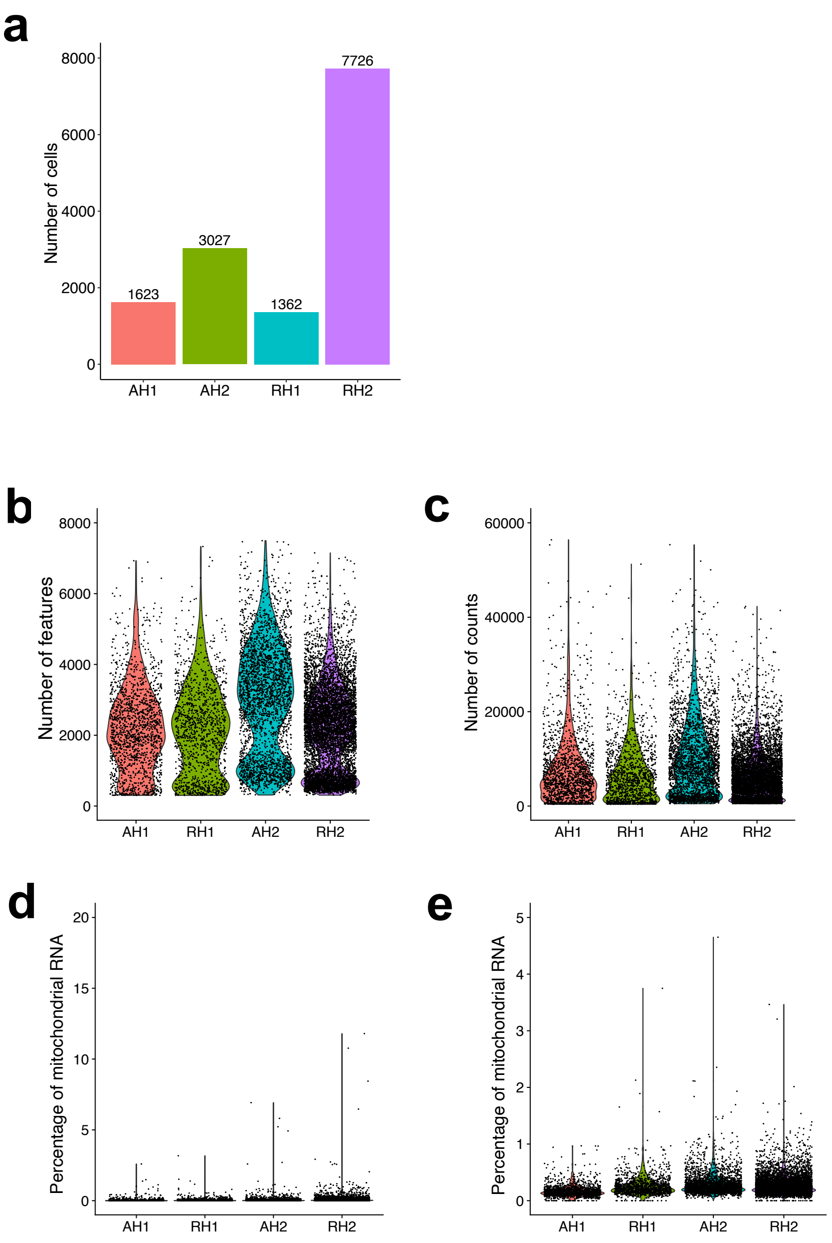

ESM Fig. 6. Post-filtering quality control metrics of cortex nuclei. (a) Number of nuclei isolated from the cortex of type 2 diabetic mice exposed to AH (AH1, AH2) or RH (RH1, RH2). Violin plots illustrating per-cell QC metrics: (b) nFeature (number of genes with at least one UMI count); (c) nCount (number of UMI counts); (d) percentage of mitochondrial genes; (e) percentage of ribosomal genes.

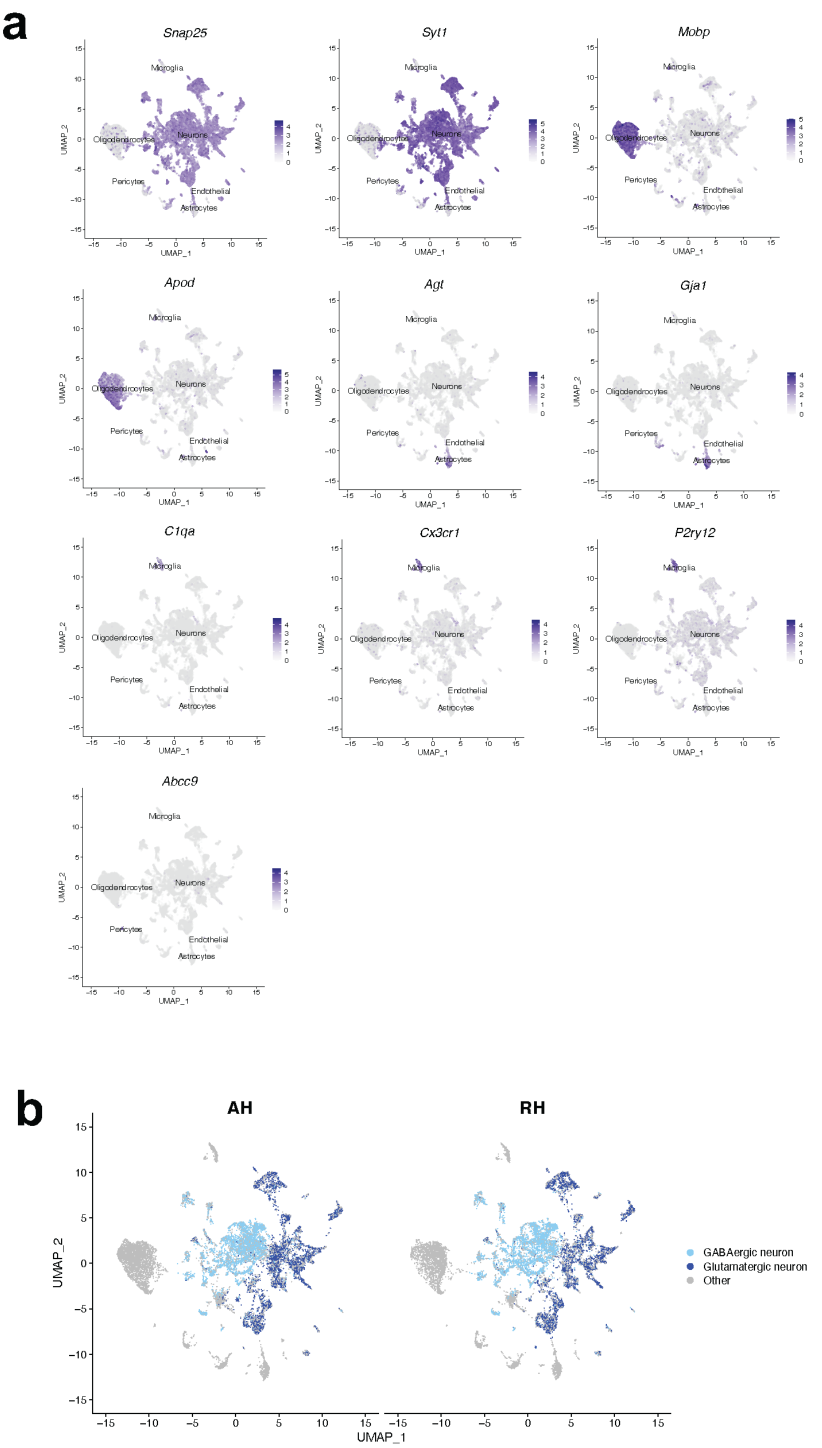

ESM Fig. 7. Annotation of cortex nuclei. UMAP plots showing the expression of canonical markers for the six cell categories (neurons, oligodendrocytes, astrocytes, microglia, endothelial cells and pericytes).

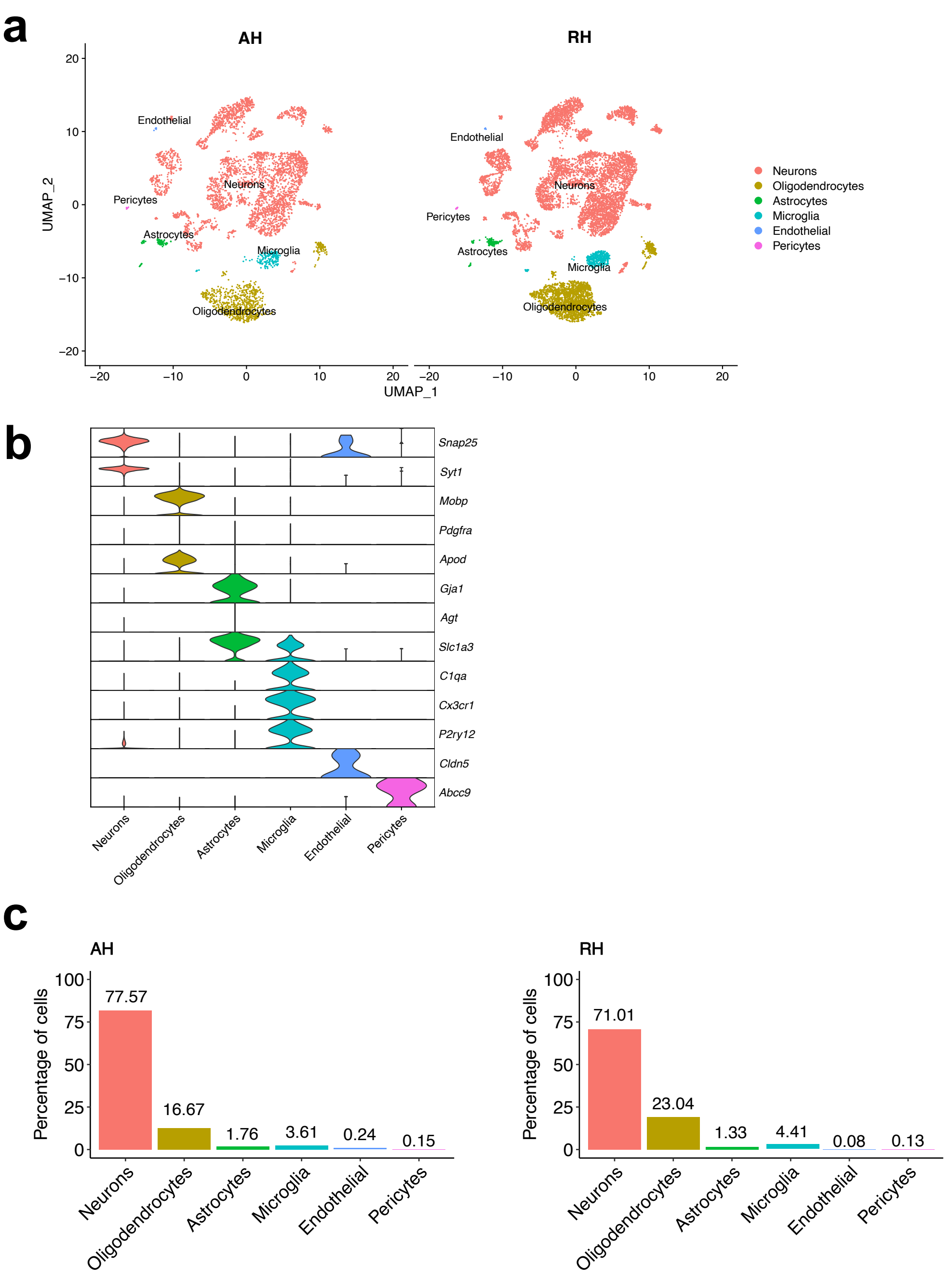

ESM Fig. 8. Clustering and annotation of cortex snRNA-seq in RH and AH type 2 diabetic mice. (a) UMAP plot showing the distribution of cortical cell types in AH and RH mice. (b) Violin plots of combined data from RH and AH showing the expression of cellular specific gene markers at each of the identified cell types. (c) Barplot depicting the percentages of the different cortical cell types per condition.

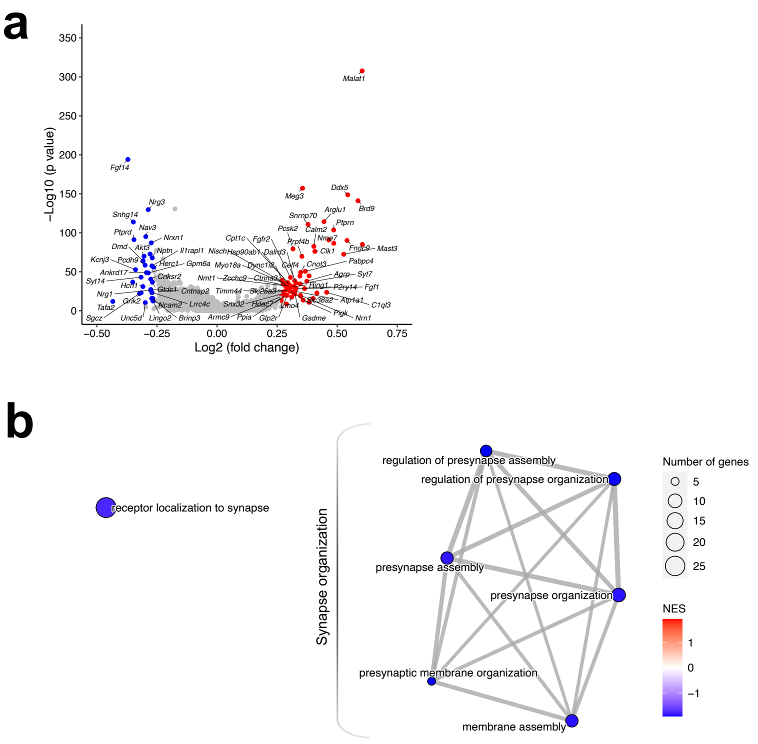

ESM Fig. 9. Transcriptional analysis of neuronal nuclei from the cortex of HFD/STZ mice exposed to AH or RH. (a) Volcano Plot depicting genes expressed in cortical neurons from mice subjected to RH as compared to AH. Red dots depict upregulated genes and blue dots depict downregulated genes (fold-change >1.2 or <-1.2 and Bonferroni adjusted P value < 0.05). Genes ending with “Rik” or starting with “Gm” were discarded from the figure. (b) Network visualization of the top enriched GO-BP terms in DE genes between RH vs AH mice. The node colour indicates the normalized enrichment score (NES), the node size indicates the number of core-enriched genes overlapping gene count and the edges represent the pairwise similarity between terms.
